# Temperature Acclimation of Chlorophyll Fluorescence and Carboxylation Capacity From Near‐Instantaneous to Weekly Time Scales

**DOI:** 10.1111/pce.70555

**Published:** 2026-04-22

**Authors:** Paul L. Drake, Erik J. Veneklaas, Nicholas G. Smith, Steven M. Driever, Hugo J. de Boer

**Affiliations:** ^1^ School of Biological Sciences The University of Western Australia Crawley Australia; ^2^ Institute of Agriculture The University of Western Australia Crawley Australia; ^3^ Department of Biological Sciences Texas Tech University Lubbock Texas USA; ^4^ Centre for Crop Systems Analysis Wageningen University Wageningen the Netherlands; ^5^ Department of Environmental Sciences Utrecht University Utrecht the Netherlands

**Keywords:** ΦPSII, carboxylation, *J*_max_, photosystem II, quantum yield, *V*_*c*max_

## Abstract

We studied near‐instantaneous temperature responses of light‐saturated photosynthesis (*A*_sat_) and the operating efficiency of photosystem II (ΦPSII) in relation to acclimation of photosynthetic biochemistry to temperature at the timescale of 1–8 days. We grew two closely related *Brassica oleracea* L. accessions under diurnal temperature ranges of 15°C–25°C and 5°C–15°C, representing the warm and cool native environments of the two accessions. Near‐instantaneous temperature responses of *A*_sat_ and ΦPSII were measured on acclimated plants exposed to a temperature ramp from 10°C to 45°C. Biochemical acclimation was quantified by switching plants between warm and cool regimes before measuring photosynthetic variables at Day 1 and Day 8 after the switch. Near‐instantaneous temperature optima of *A*_sat_ and ΦPSII functionally shifted towards the acclimated growth temperatures in both accessions, and both accessions achieved their highest instantaneous maximum values of *A*_sat_ and ΦPSII when grown under representative native regimes. Differences in maximum carboxylation capacity were evident by Day 1; no further change was detected by Day 8. Our results highlight functional coordination between adaptation, acclimation and near‐instantaneous temperature responses of photochemistry and biochemistry. Moreover, our results suggest photosynthetic biochemistry can acclimate to temperature changes within a day.

## Summary statement

Thermal acclimation of net photosynthesis, photochemistry and carboxylation was studied in *Brassica oleracea* accessions. Net photosynthesis and photochemistry functionally adapted and acclimated to temperature. Carboxylation acclimated within 1 day, much faster than is generally assumed.

## 1 Introduction

Plants exhibit genotypic adaptation of the photosynthetic apparatus to the temperature regimes they evolved under, and many species also undergo phenotypic thermal acclimation of photosynthetic components over shorter timeframes (Berry and Bjorkman 1980; Miyazawa and Kikuzawa 2006; Kattge and Knorr 2007; Yamori et al. 2014; Kumarathunge et al. 2019). The mechanisms responsible for the biochemical adaptation and acclimation of net photosynthesis to temperature relate to changes in the quantity and reaction rate of the enzymes that perform photosynthesis and respiration, adjustment to the pools of carbon cycle intermediates and, for C_3_ species, shifts in the solubility ratio and enzyme specificity of CO_2_ and O_2_, which leads to changes in the degree of inhibition of photosynthesis due to photorespiration (Hall and Keys 1983; Brooks and Farquhar 1985; Salisbury and Ross 1985; Campbell et al. 2007; Sage and Kubien 2007; Yamori et al. 2014; Dusenge et al. 2019).

There is also evidence of photochemical temperature acclimation specific to the whole‐chain electron transport rate (ETR) and the photosystem II (PSII) ETR (Mitchell and Barber 1986; Mawson and Cummins 1989; Yamori et al. 2008; Vico et al. 2019). However, there is little specific empirical knowledge on the timescales at which biochemical and photochemical processes respond to temperature change. Given the current rate of atmospheric warming owing to human activities (IPCC 2022) and the increased risk of temperature extremes associated with this warming (Coumou and Robinson 2013, Viovy et al. forthcoming), it is imperative that the processes underlying photosynthetic temperature adaptation and acclimation are resolved further.

Despite theoretical advances and empirical observations of the instantaneous temperature response of net photosynthesis, we are yet to fully understand the processes and timeframes underlying thermal adaptation and acclimation. Take, for example, the maximum carboxylation rate of ribulose‐1,5‐bisphosphate (RuBP) by ribulose‐1,5‐bisphosphate carboxylase‐oxygenase (Rubisco) (termed *V*_*c*max_). The acclimation of *V*_*c*max_ to the environment is achieved by altering the form and amount of Rubisco in the leaf, and the half‐life of Rubisco is approximately 1 week (Simpson et al. 1981). Based on this half‐life, an acclimation timeframe of 1–2 weeks for *V*_*c*max_ is conceivable (Yamori et al. 2006; Smith and Dukes 2017; Ren et al. 2024). However, the specific site for the temperature inhibition of Rubisco is Rubisco activase (Feller et al. 1998; Haldimann and Feller 2004), and some forms of this enzyme are able to respond to heat stress within 4 h (Law and Crafts‐Brandner 2001; Yamori et al. 2014). Furthermore, Scafaro et al. (2023) effectively modelled the acclimation response of photosynthesis to high temperature by accounting for Rubisco deactivation, a process that can occur on a timeframe of minutes to hours. These potential differences point to the need for further research to help establish broad empirical support for the acclimation timeframe of photosynthesis, and for Rubisco activity in particular. Because the rate‐limiting steps of photosynthesis can shift from Rubisco activity to electron transport depending on temperature, it is essential to evaluate both processes together.

Theory and empirical data suggest that the potential rate of electron transport for the regeneration of RuBP (*J*_max_) scales in a predictable fashion with *V*_*c*max_ (Wullschleger 1993; Medlyn et al. 2002; Kattge et al. 2009). Nonetheless, despite this general scaling relationship, *J*_max_ and *V*_*c*max_ are differentially sensitive to temperature (Medlyn et al. 2002; Kattge and Knorr 2007). Given the dependency of the regeneration of RuBP on products of the electron transport chain (namely, ATP and NADPH), it is likely that, despite the need for overall coordination, the thermal limitation and acclimation capacity of electron transport differ from Rubisco activity. In some situations, this difference may yield unrealistic predictions from photosynthetic models that focus on the mechanistic expression of Rubisco, such as the seminal Farquhar, von Caemmerer and Berry (FvCB) model of C_3_ photosynthesis (Farquhar et al. 1980). This has been addressed mechanistically for Rubisco (Collatz et al. 1991) and empirically for electron transport via chlorophyll fluorescence (van der Tol et al. 2014), but the predictive range of these model variants is limited.

An important chlorophyll fluorescence parameter to help probe the performance of the electron transport system in relation to the environment is the operating efficiency of PSII photochemistry, termed ΦPSII (Genty et al. 1989; Maxwell and Johnson 2000). ΦPSII represents the proportion of light absorbed by the leaf that is used in PSII photochemistry, which can be resolved to the PSII ETR (Murchie and Lawson 2013). The PSII ETR may be downregulated due to photodamage to the reaction centres or as a result of non‐photochemical quenching, which can occur at temperature extremes (Schreiber and Berry 1977; Berry and Bjorkman 1980; Porcar‐Castell 2011); these are processes that can reduce net photosynthesis (Havaux 1992; Oberhuber and Edwards 1993). However, this effect may be partly offset by greater whole‐chain ETR owing to increased fluidity of the thylakoid membranes with increasing temperature (Yamori et al. 2008). Furthermore, at moderately high temperatures, PSII can rapidly acclimate, with response times on the order of 30 min to 2 h (Havaux 1993; Zhu et al. 2024). Despite this level of mechanistic understanding, the interrelationship of the temperature response of electron transport and photosynthetic biochemistry, and its effect on net photosynthesis, has not been fully resolved. Moreover, in general, the temperature response and potential for acclimation of ΦPSII are not considered when estimating *V*_*c*max_ and *J*_max_, such as within the FvCB model framework, as it is usually assumed to be constant. This assumption may affect the predictive capability of crop models and land surface models, particularly when photosynthesis is estimated above or below the thermal optima (Rogers et al. 2017).

Although there is a widely accepted scheme for the adaptation and acclimation of the light‐saturated net photosynthetic rate (*A*_sat_) in relation to thermal regimes, there is currently no equivalent scheme for ΦPSII. Using two closely related *Brassica oleracea* L. accessions, we address this knowledge gap, and test for coordination between the thermal acclimation of *A*_sat_ and ΦPSII. Furthermore, using the same accessions, we also test the generalised assumption that carboxylation capacity acclimates to a new thermal regime on a timescale of approximately 1 week. To our knowledge, this is the first systematic empirical test of the acclimation timeframe of *V*_*c*max_ in relation to temperature. Our specific Hypotheses were that (1) both ΦPSII and *A*_sat_ will functionally adapt and acclimate to thermal regimes, and the acclimation response of these two variables will be well coordinated, and (2) consistent with the half‐life of Rubisco, the acclimation timeframe of *V*_*c*max_ will be approximately 1 week.

To address these hypotheses, we measured the near‐instantaneous temperature response of net photosynthesis and ΦPSII in plants acclimated to the experimental warm and cool temperature regimes. We define the ‘near‐instantaneous’ responses as those occurring within a timeframe of minutes. Moreover, our experimental setup and measurement regime considered the temperature acclimation timescale of *V*_*c*max_ and *J*_max_ to an instantaneous switch between warm and cold temperature regimes.

## 2 Materials and Methods

### 2.1 Plant Material

Seeds of two *B. oleracea* L. accessions were sourced from a genetic population development programme overseen by the Commonwealth Scientific and Industrial Research Organisation in Perth, Western Australia. Accession CAF006 originates from Zimbabwe (Chikukwa Commune, Manicaland). The mean maximum daily temperature and the maximum daily temperature record for the period 1975 to 2024 at a representative weather station (Chimoio, Zimbabwe: −19.117°, 33.467°) were 26.7°C and 40.5°C, respectively (NOAA 2024). Based on its origin and the observation that this accession flowers without vernalisation, CAF006 is regarded as being adapted to relatively warm climates and is henceforth referred to as ‘CAF‐Warm’. Accession CAF0216 originates from Spain, near La Coruña in the Galicia region. The mean maximum daily temperature and the maximum daily temperature record for the period 1975 and 2024 at a representative weather station (A Coruña, Spain: 43.3669°, −8.4192°) were 17.9°C and 34.4°C, respectively (NOAA 2024). Accession CAF0216 flowers only after vernalisation and is regarded as typical of wild *B. oleracea*, which are generally adapted to cool climates, require vernalisation and are biennial. Based on this information, accession CAF0216 is referred to as ‘CAF‐Cool’ hereon. The advantage of selecting these closely related accessions originating from different climates is to remove potential ambiguities associated with phylogenetic effects.

### 2.2 Growth Rooms and Temperature Regimes

Plants were germinated and grown in two controlled environmental rooms. During the growth phase of the experiments described below, plants experienced two air temperature (*T*_air_) regimes: 15°C:25°C (night:day), termed the ‘H’ regime, and 5°C:15°C (night:day), termed the ‘L’ regime. All other variables of the growth rooms were identical (see details below). Within each growth room, plants were grown in a completely randomised design, and the plants were re‐randomised each week. Notwithstanding differences in air temperature regimes, the environmental conditions of the growth rooms were approximately equivalent (Figure S1). However, the temperature effects observed in our study are limited to the conditions of the present growth room settings.

Experiment 1 Near‐instantaneous temperature responses of ΦPSII and A_sat_.

Seeds of both *B. oleracea* accessions were germinated on moist filter paper in petri dishes under the following conditions: atmospheric CO_2_ (*C*_a_): ambient (400–420 µmol mol^−1^), *T*_air_: 15°C:25°C (night:day), daytime photosynthetically active radiation (PAR): 700 µmol m^−1^ s^−1^, night:day photoperiod: 14:10 h, relative humidity (RH): set to be > 50% (although the RH inside the petri dishes was close to 100%)—the actual range in RH is shown in Figure S1. The day–night temperature transitions occurred at a rate of 4 min/1°C of change in *T*_air_. One week after germination, 10 seedlings from each accession were transferred to 2.8 L pots filled with an organic potting mix (50% river sand, 25% pine bark, 25% peat moss) and well‐watered to encourage establishment (all other environmental variables were the same as during germination). After 1 week of establishment in the pots, half of the plants from each accession were transferred to a *T*_air_ of 5°C:15°C (night:day), that is, the L regime, all other environmental variables were equivalent to those of the establishment phase. Thus, for each accession, five plants were grown in the 15°C:25°C growth temperature treatment (H regime) and five plants were grown in the 5°C:15°C growth temperature treatment (L regime). Note that by holding RH at or above 50% and varying *T*_air_, the atmospheric vapour pressure deficit (VPD, kPa) of growth rooms differed. For example, the average daytime VPD of 15°C:25°C room was 1.4 kPa, whereas the average daytime VPD of the 5°C:15°C room was 0.5 kPa.

**Figure 1:**
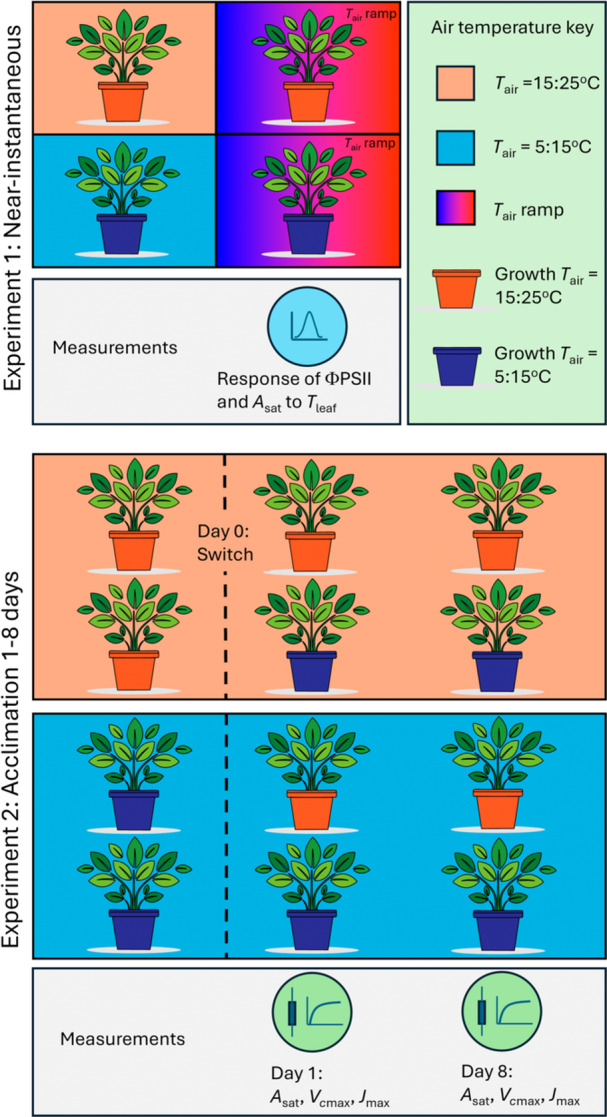
The approach for *Experiment 1* involved growing plants from two *B. oleracea* accessions: one originating from a warm climate, the other originating from a cool climate. Plants were grown under specific air temperature (*T*_air_) regimes (*T*_air_: 15°C:25°C night:day, also termed the H regime, or 5°C:15°C night:day, also termed the L regime). After plants developed under these conditions, the near‐instantaneous responses of the operating efficiency of photosystem II (ΦPSII) and the light‐saturated photosynthetic rate (*A*_sat_) to leaf temperature (*T*_leaf_) were determined. To capture the near‐instantaneous response, plants were exposed to a *T*_air_ ramp of 15°C–35°C, with *T*_leaf_ regulated to a range of approximately 10°C–45°C using the temperature control of a gas exchange system. For *Experiment 2*, plants were grown under the same conditions as *Experiment 1*, but after the same period of development half of the plants of each *T*_air_ regime were switched to the opposing temperature regime (Day 0: Switch). *A*_sat_, the apparent maximum rate of carboxylation (*V*_*c*max_) and the apparent maximum rate of electron transport (*J*_max_) were determined 1 and 8 days after the switch. Plants within each growth temperature treatment were of an equivalent age at the time of the near‐instantaneous response measurements of *Experiment 1* and at 1 day after the switch of *Experiment 2*.

Each week, 50 mL of a commercial complete fertiliser (Thrive, All Purpose Soluble Fertiliser, N:P:K 25:5:8.8) was applied to each pot. The first application was at a concentration of 1.5 g L^−1^; subsequent applications were at a concentration of 3 g L^−1^. Plants were well‐watered under these conditions for 21 days of growth and 41 days of growth for the 15°C:25°C and 5°C:15°C growth temperature treatments, respectively, before gas exchange and fluorescence measurements were implemented. The additional time for growth in the 5°C:15°C group ensured that all plants within an accession were approximately the same height prior to measurements (12–15 cm for CAF‐Warm and 10–14 cm for CAF‐Cool). For plants of each temperature regime, a fully expanded leaf that was three or four leaves back from the apex was selected for gas exchange measurements. The experimental approach is outlined in Figure 1.

### 2.3 The Operating Efficiency of Photosystem II and Leaf Gas Exchange

The operating efficiency of photosystem II (ΦPSII) was measured on each plant (*n* = 1 leaf/plant) with a pulse‐amplitude modulated fluorometer (Model LI‐6800, LI‐COR Environmental, USA). Simultaneous measurements of the steady‐state light‐saturated photosynthetic rate (*A*_sat_, μmol m^−2^ s^−1^) and stomatal conductance to water vapour (*g*_sw_, mol H_2_0 mol^−1^ air) were taken with the same instrument, with ΦPSII and *A*_sat_ measured after *g*_sw_ had stabilised. The following fluorometer settings were implemented: dark modulation rate: 500 Hz, light modulation rate 1 kHz, flash modulation rate 250 kHz, averaging time: 15 s, setpoint: 100 µmol m^−2^ s^−1^, rectangular flash, red target: 8000 µmol m^−2^ s^−1^ and duration: 1000 ms. The environmental variables of the leaf chamber during which *g*_sw_ stabilised were as follows: PAR: 1500 µmol m^−2^ s^−1^ (colour spec: r90b10), *C*_a_: 400 µmol CO_2_ mol^−1^ air and RH: 40%–70%. Note that ΦPSII was thus measured from leaves exposed to a PAR of 1500 µmol m^−2^ s^−1^, representing a realistic operational value, and not a maximum value. Gas exchange variables and ΦPSII were measured across a range of temperate, spanning a leaf temperature (*T*_leaf_,°C) of approximately 10°C–45°C at a 5°C interval. To achieve this temperature range, three growth rooms were used alongside the temperature control system of the instrument. The following environmental variables were set for each room: *C*_a_: ambient (400–420 µmol CO_2_ mol^−1^ air), PAR: 700 µmol m^−2^ s^−1^, RH: 50%, *T*_air_: 15°C (Room 1), 25°C (Room 2), and 35°C (Room 3).

The same leaf was used for measurements across the temperature range, with measurements taken over a 10‐h daytime photoperiod. The order of measurements generally began with temperatures at or near the growth environment of a specific plant and finished with the highest *T*_leaf_ in the sequence (35°C–45°C). Measuring ΦPSII at the highest *T*_leaf_ at the end of the sequence ensured that plants were not exposed to a prolonged period of elevated *T*_air_ prior to taking further measurements. Between measurements, plants were returned to the *T*_air_ at which they grew. In relation to *T*_leaf_, the measurement sequence for plants grown at 15°C:25°C was: 25°C, 30°C (Room 2), 15°C, 20°C, 10°C (Room 1), 35°C, 40°C, 45°C (Room 3). For plants grown at 5°C:15°C, the *T*_leaf_ measurement sequence was: 15°C, 20°C, 10°C (Room 1), 25°C, 30°C (Room 2), 35°C, 40°C, 45°C (Room 3).

From the measurements, the near‐instantaneous response of ΦPSII to *T*_leaf_ and *A*_sat_ to *T*_leaf_ was described by a rectangular function (Neri et al. 2024):

(1) $$\Phi \text{PSII or}\;A_{\text{sat}}=f\left(T_{\text{leaf}}\right)=a0.5\left(\text{erf}\left(\frac{T_{\text{leaf}}-m_{1}}{s_{1}}\right)+\text{erf}\left(\frac{m_{2}-T_{\text{leaf}}}{s_{2}}\right)\right),$$

where erf is the error function with a sigmoidal form over the interval 0, *z*:

(2) $$\text{erf}\left(z\right)=\frac{2}{\sqrt{\pi }}\int_{0}^{z}e^{{-t}^{2}}dt,$$

where *t* is the integration variable.

The rectangular function was used as an empirical model to describe the relationships between ΦPSII and *A*_sat_ with *T*_leaf_. The function was selected because it performed well across the entire range of *T*_leaf_, which was typically characterised as an asymmetric response. Equation (1) was fit to the empirical data set of each plant using the Levenberg–Marquardt algorithm iterative procedure (Levenberg 1944; Marquardt 1963) using OriginPro Version 2024b (OriginLab Corporation, Northampton, MA, USA). Although Neri et al. (2024) use Equation (1) to describe relationships between the maximum yield of ΦPSII and *T*_air_, the parameters have functional relevance to the responses of ΦPSII and *A*_sat_ to *T*_leaf_. In our study, *a* is the maximum ΦPSII or *A*_sat_ without limitations imposed by temperature, *m*_1_ (*m*_2_) is the *T*_leaf_ at which ΦPSII/*A*_sat_ is 50% of *a* and *s*_1_ (*s*_2_) represents the slope of peak change in ΦPSII/*A*_sat_ at *m*_1_ (*m*_2_). In addition to these parameters, we also define the maximum ΦPSII (ΦPSII_[max]_) and maximum *A*_sat_ (*A*_sat[max]_) over the range of measured *T*_leaf_ and the *T*_leaf_ at ΦPSII_[max]_ and *A*_sat[max]_ (*T*_opt [ΦPSII]_ and *T*_opt [*A*sat]_, respectively). We also determined the ΦPSII and *A*_sat_ at a fixed *T*_leaf_ of 40°C, termed ΦPSII_[40]_ and *A*_sat[40]_ for ΦPSII and *A*_sat_, respectively. This *T*_leaf_ was selected because it represents a realistic temperature extreme for these accessions during a growing season. The relative difference between ΦPSII_[max]_ and ΦPSII_[40]_ (ΔΦPSII) and the relative difference between *A*_sat[max]_ and *A*_sat[40]_ (Δ*A*_sat_) were also determined. These derived parameters are depicted in Figure S2.

Experiment 2 Acclimation of V_cmax_ and J_max_ from 1 to 8 days.

Seeds were germinated and plants were established in the same manner as *Experiment 1*. As with *Experiment 1*, after 1 week of establishment in the pots, half of the plants from each accession were transferred to a *T*_air_ of 5°C:15°C (night:day), that is, the L regime, all other environmental variables were equivalent to those of the establishment phase. For this experiment, 10 plants were grown in the 15°C:25°C growth temperature treatment (H regime) and 10 plants were grown in the 5°C:15°C growth temperature treatment (L regime). As per *Experiment 1*, 50 mL of a commercial complete fertiliser (Thrive, All Purpose Soluble Fertiliser, N:P:K 25:5:8.8) was applied to each pot each week. The first application was at a concentration of 1.5 g L^−1^; subsequent applications were at a concentration of 3 g L^−1^.

At the same developmental phases as described for measurements in *Experiment 1*, plants of *Experiment 2* received a short‐term acclimation treatment. The short‐term acclimation treatment involved transferring half the plants from each growth temperature into the opposing growth temperature during the morning (1–2 h after the daytime photoperiod had been initiated). Plants that remained at their growth temperature regimes served as controls. The experimental design and measurement protocol are shown in Figure 1. The measurement protocol is also described in detail below. Our experimental design aimed to identify possible differences in photosynthetic parameters owing to genotypic adaptation (between accession differences), as well as differences due to acclimation during plant growth and differences due to short‐term acclimation following the transfer step described above.

### 2.4 Leaf Gas Exchange Properties

Leaf gas exchange properties were measured with an open‐flow photosynthesis system (Model LI‐6400XT, LI‐COR Environmental, USA) 1 day after the short‐term acclimation treatment was implemented. *A*_sat_ was measured on each plant (*n* = 1 leaf/plant) under the following leaf chamber conditions: *C*_a_: 400 µmol CO_2_ mol^−1^ air, PAR: 1500 µmol m^−2^ s^−1^, RH: 50%–70%, *T*_leaf_: 25°C or 15°C (equivalent to the *T*_air_ of each plant growth environment at the time of measurement). The response of *A*_sat_ to intercellular CO_2_ (*C*_i_, µmol CO_2_ mol^−1^ air) was derived by varying *C*_a_ inside the leaf chamber over the following stepwise sequence: 400, 300, 200, 100, 50, 400, 600, 900, 1200, 2000 µmol CO_2_ mol^−1^ air, with other chamber variables remaining the same as the initial *A*_sat_ measurement. All gas exchange measurements were taken after *A*_sat_ and *g*_s_ stabilised. Measurements of *A*_sat_ and the response of *A*_sat_ to *C*_i_ were repeated 8 days after the short‐term acclimation treatment was implemented.

A biochemical model of C_3_ photosynthesis (Farquhar et al. 1980) was fit to the relationships between *A*_sat_ and *C*_i_ using the fitaci function within the ‘plantecophys’ package (Duursma 2015) in R, version 4.4.1 (R Development Core Team 2025), using the default fitting method and allowing dark respiration to be estimated during fitting. We also applied the default assumption that mesophyll conductance is infinite and we maintained the default temperature dependencies of the CO_2_ compensation point in the absence of mitochondrial respiration and the Michaelis–Menten constants for CO_2_ and O_2_ from Bernacchi et al. (2001). The apparent maximum rate of carboxylation and the apparent maximum ETR were determined at the measurement *T*_leaf_, corresponding to the daytime *T*_air_ of each growth room (hereafter termed *V*_*c*max_ and *J*_max_, respectively) and at a standardised *T*_leaf_ of 25°C (*V*_*c*max25_ and *J*_max25_, respectively), with standardisation conducted within the ‘plantecophys’ package using a peaked Arrhenius equation from Medlyn et al. (2002). In theory, the photosynthetic variables measured at *T*_leaf_ incorporate the effect of temperature on enzymatic kinetics, whereas the temperature‐corrected photosynthetic variables represent the maximum carboxylation capacity and the maximum electron transport capacity. However, we applied caution when interpreting the temperature‐corrected variables owing to the potential for species/accession‐specificity for the fitting parameters of the peaked Arrhenius equation.

We also note the potential for temperature measurement errors in the Li6400XT (Garen et al. 2022). However, we set *T*_leaf_ to the same value as the respective growth room *T*_air_ when deriving relationships between *A*_sat_ and *C*_i_, with the resulting difference between the *T*_air_ of the growth room and the *T*_air_ of the instrument ranging from −1.3°C to 4.5°C, with a median difference of 0.9°C across all measurements. Differences between the *T*_air_ of the growth rooms and the *T*_air_ of the LI‐6800 during near‐instantaneous responses (*Experiment 1*) were larger, ranging from −18.4°C to 5.0°C. However, the potential for artifacts associated with temperature measurements is much smaller for this instrument (Garen et al. 2022).

### 2.5 Statistical Analysis

Statistical analyses were undertaken in R version 4.4.1 (R Development Core Team 2025). Parametric analyses were preceded by tests for normality (Shapiro–Wilk tests (Shapiro and Wilk 1965)), with Yeo–Johnson transformations (Yeo 2000) and Box–Cox transformations (Box and Cox 1964) applied to achieve normality as needed. Because each temperature regime was implemented in a single growth room, statistical inference for temperature is conditional on the present chamber settings; therefore, we interpret temperature‐related tests as differences between regimes under these conditions.

Two‐way ANOVAs and Tukey's post hoc comparisons were used to test for differences in the variables associated with the near‐instantaneous temperature responses of ΦPSII and *A*_sat_, with significant differences defined at an *α* level of 0.05. In each case, accession and growth temperature were treated as between‐group factors, and an interaction between accession and growth temperature was included.

Linear mixed effect models were used to test for differences between accession, growth temperature and acclimation day using the ‘nlme’ package (Lindstrom and Bates 1990; Pinheiro et al. 2026). The following response variables were tested in the models: *V*_*c*max_, *J*_max_, *J*_max_/*V*_*c*max_, *V*_*c*max25_, *J*_max25_ and *V*_*c*max25_/*J*_max25_ with accession and growth temperature treated as between‐group factors, acclimation day treated as a within‐group factor and each individual plant treated as a random intercept term; interaction terms between accession and acclimation temperature, acclimation day and accession, acclimation day and acclimation temperature and acclimation day, acclimation temperature and accession were included in the models. Tukey pairwise comparisons of the model outputs were then determined using the ‘emmeans’ package.

Standardised major axis (SMA) regression analyses were implemented to describe relationships between traits using the ‘smatr’ package (Warton et al. 2012), including relationships between *A*_sat_ and *V*_*c*max_ and *A*_sat_ and *J*_max_. Data were aggregated for fitting based on tests for significance between the slope and intercept of accession by treatment groupings. Where appropriate, the significance of relationships was assessed further using repeated measures correlation tests from the ‘rmcorr’ package (Bakdash and Marusich 2017).

## 3 Results

Experiment 1 Near‐instantaneous temperature responses of ΦPSII and A_sat_.

**Figure 2:**
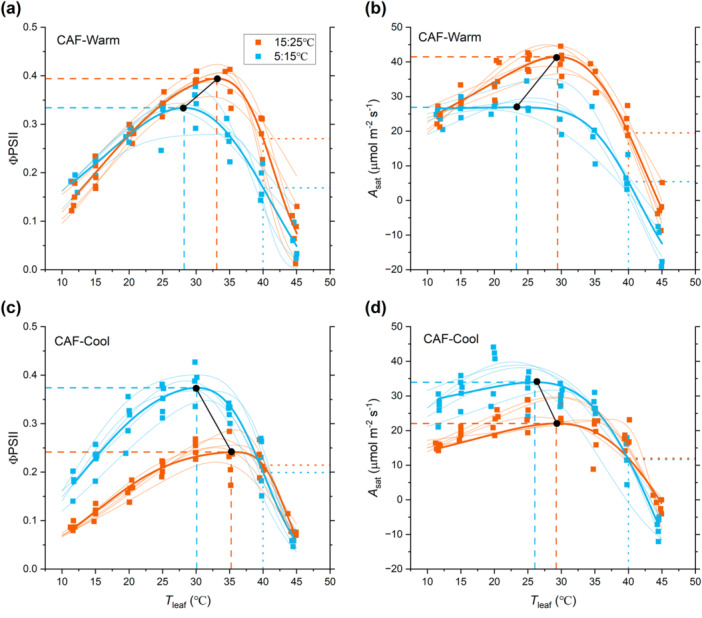
Near‐instantaneous responses of ΦPSII to *T*_leaf_ (a and c) and *A*_sat_ to *T*_leaf_ (b and d) for each accession and growth temperature. Fitted lines are rectangular models. Thin lines represent models for each plant. Thick lines are the overall models for each accession by temperature regime. The dashed lines depict the overall optimal temperature for ΦPSII and *A*_sat_ (*T*_opt[ΦPSII]_ and *T*_opt[*A*sat]_, respectively) and the overall maximum ΦPSII and *A*_sat_ (ΦPSII_[max]_ and *A*_sat[max]_, respectively). The solid black lines show the difference between ΦPSII_[max]_ and *A*_sat[max]_ for the warm‐grown and cool‐grown plants of each accession. The dotted lines represent the overall ΦPSII and *A*_sat_ at 40°C (ΦPSII_[40]_ and *A*_sat[40]_, respectively). Analysis of accession by treatment differences of these parameters, along with the relative difference between ΦPSII_[max]_ and ΦPSII_[40]_, and *A*_sat[max]_ and *A*_sat[40]_ (ΔΦPSII and Δ *A*_sat_, respectively) are shown in Table 1. [Color figure can be viewed at wileyonlinelibrary.com]

**Table 1:** ANOVA test results for differences in *T*_opt[ΦPSII]_, *T*_opt[*A*sat]_, ΦPSII_[max]_, *A*_sat[max]_, ΦPSII_[40]_, *A*_sat[40]_, ΔΦPSII and Δ*A*_sat_ based on accession and growth regime (*T*_air_ = 15°C:25°C vs. 5°C:15°C).

Variable	Test	DF	Sum of Sq	*F*	*p*
*T*_opt[ΦPSII]_	Accession	1	5.69	1.69	0.213
	Growth regime	1	79.11	23.54	**< 0.001** ***
	Accession × Growth regime	1	0.42	0.13	0.728
	Model	3	84.23	8.36	**< 0.001** ***
ΦPSII_[max]_	Accession	1	0.01	14.98	**0.002** **
	Growth regime	1	0.01	4.88	**0.043** *
	Accession × Growth regime	1	0.04	54.77	**< 0.001** ***
	Model	3	0.06	26.40	**< 0.001** ***
*T*_opt[*A*sat]_	Accession	1	5.19	0.54	0.474
	Growth regime	1	139.78	14.54	**0.002** **
	Accession × Growth regime	1	4.25	0.44	0.516
	Model	3	150.43	5.22	**0.011** *
*A*_sat[max]_	Accession	1	91.70	7.17	**0.017** *
	Growth regime	1	0.82	0.06	0.803
	Accession × Growth regime	1	582.07	45.48	**< 0.001** ***
	Model	3	703.39	18.32	**< 0.001** ***
ΦPSII_[40]_	Accession	1	< 0.01	0.60	0.452
	Growth regime	1	0.01	6.22	**0.025** *
	Accession × Growth regime	1	0.01	7.77	**0.014** *
	Model	3	0.02	4.79	**0.016** *
*A*_sat[40]_	Accession	1	0.76	0.05	0.823
	Growth regime	1	589.20	40.18	**< 0.001** ***
	Accession × Growth regime	1	110.87	7.56	**0.015** *
	Model	3	673.46	15.31	**< 0.001** ***
ΔΦPSII	Accession	1	0.02	1.95	0.183
	Growth regime	1	0.18	15.25	**0.001** **
	Accession × Growth regime	1	0.01	0.54	0.475
	Model	3	0.21	5.89	**0.007** **
Δ*A*_sat_	Accession	1	0.07	3.72	0.073
	Growth regime	1	0.60	32.81	**< 0.001** ***
	Accession × Growth regime	1	< 0.01	< 0.01	0.978
	Model	3	0.65	11.86	**< 0.001** ***

*Note:* Accession by treatment differences are also depicted graphically in Figures S5 and S6. Boldface indicates a significant difference.

* 0.01 ≤ *p* ≤ 0.05;

** 0.001 ≤ *p* < 0.01;

*** *p* < 0.001.

The near‐instantaneous temperature responses of ΦPSII and *A*_sat_ for each accession are shown in Figure 2. The model fitting parameters and goodness of fit for each plant are shown in Table S1, with the first derivative of the fitted functions shown in Figure S3. Note that across the temperature range, there was no clear response of *g*_sw_ (Figure S4 and Table S2). From Figure 2, distinct differences in the response functions are evident. For example, the maximum ΦPSII and the maximum *A*_sat_ (ΦPSII_[max]_ and *A*_sat[max]_, respectively), were higher in warm‐grown plants compared to cool‐grown plants of accession CAF‐Warm (Figure 2a,b and Figure S5c,d). In contrast, ΦPSII_[max]_ and *A*_sat[max]_ were higher in cool‐grown plants compared to warm‐grown plants of accession CAF‐Cool (Figure 2c,d and Figure S5c,d). A two‐way ANOVA conferred a significant interaction between accession and growth regime (*T*_air_ = 15°C:25°C vs 5:15°C) for both ΦPSII_[max]_ and *A*_sat[max]_ (Table 1, *p* < 0.001). The temperature response pattern was different for the thermal optima of ΦPSII and *A*_sat_ (*T*_opt[ΦPSII]_ and *T*_opt[*A*sat]_, respectively). In this case, *T*_opt[ΦPSII]_ and *T*_opt[*A*sat]_ were greater in warm‐grown plants of both accessions (Figure 2 and Figure S5a,b) and there was no significant interaction between accession and growth regime for either response variable (Table 1, *p* > 0.05).

**Figure 3:**
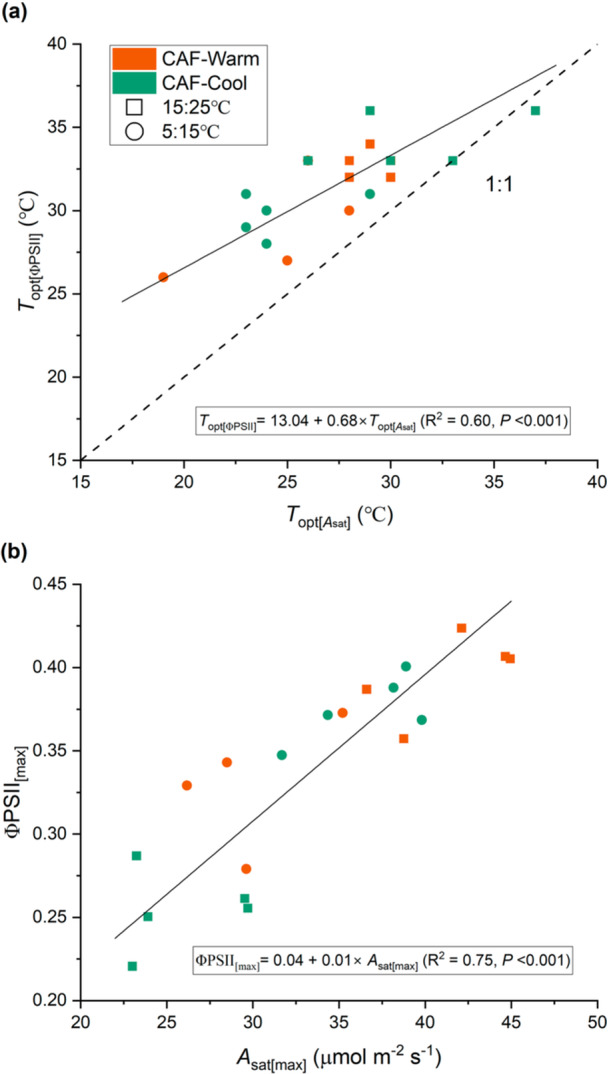
Relationships between *T*_opt[ΦPSII]_ and *T*_opt[*A*sat]_ (a), and ΦPSII_[max]_ and *A*_sat[max]_ (b). Fitted lines are SMA regression models. Dashed line in (a) is the 1:1 ratio. The near‐instantaneous temperature responses used to derive the variables of the figure were measured over a *T*_leaf_ range of approximately 10°C to 45°C. The growth temperatures of the plants are indicated by the shape of the points: 15°C:25°C and 5°C:15°C. [Color figure can be viewed at wileyonlinelibrary.com]

On average, *T*_opt[ΦPSII]_ and *T*_opt[*A*sat]_ were 4.3°C and 5.4°C greater, respectively, for plants grown at 15°C:25°C compared to those grown at 5°C:15°C. *T*_opt[ΦPSII]_ and *T*_opt[*A*sat]_ were positively correlated, with the slope of the relationship significantly different from 1 (slope = 0.70°C/°C, lower limit = 0.51°C/°C, upper limit = 0.96°C/°C) and the intercept significantly different from 0 (intercept = 12.5°C, lower limit = 6.22°C, upper limit = 18.89°C) (Figure 3a). Thus, for a given *T*_opt[*A*sat]_, *T*_opt[ΦPSII]_ was greater than *T*_opt[*A*sat]_, but there was a tendency for *T*_opt[ΦPSII]_ and *T*_opt[*A*sat]_ to converge at the upper limit of the observed values, and this part of the bivariate relationship was dominated by data obtained from plants grown at 15°C:25°C. ΦPSII_[max]_ and *A*_sat[max]_ were also positively correlated, with a 0.01 increase in ΦPSII_[max]_ for every 1 µmol m^−2^ s^−1^ increase in *A*_sat[max]_ (Figure 3b).

Further analysis of the temperature response of ΦPSII and *A*_sat_ with specific consideration of the temperature range above the observed optima, or that experienced during growth, is shown in Figure S6 and Table 1. ΦPSII_[40]_ and *A*_sat[40]_ were higher in warm‐grown plants of accession CAF‐Warm, but there was no difference in these variables due to growth regime for accession CAF‐Cool (Figure S6a,b and Table 1). Thus, for accession CAF‐Warm, growth at 15°C:25°C resulted in a higher ΦPSII and *A*_sat_ at 40°C compared to growth at 5°C:15°C, but this photosynthetic adjustment for warm‐grown plants at 40°C was absent for accession CAF‐Cool. In contrast, ΔΦPSII and Δ*A*_sat_ were more negative in cool‐grown plants of both accessions and overall, Δ*A*_sat_ was 1.6‐fold more negative than ΔΦPSII for warm‐grown plants and Δ*A*_sat_ was 1.7‐fold more negative than ΔΦPSII for cool‐grown plants (Figure S6b,c and Table 1). Hence, for both accessions, there was a greater relative photosynthetic reduction between the optima (ΦPSII_[max]_ and *A*_sat[max]_) and the value at 40°C (ΦPSII_[40]_ and *A*_sat[40]_) in cool‐grown plants, and the relative reduction was greater for *A*_sat_ than for ΦPSII.

Experiment 2 Acclimation of V_cmax25_ and J_max25_ from 1 to 8 days.

**Figure 4:**
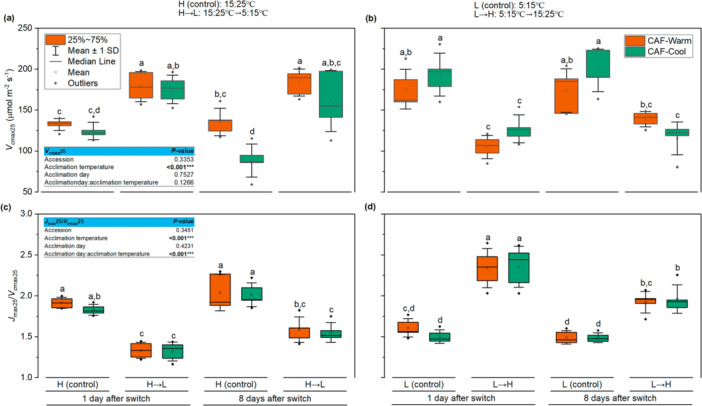
*V*_*c*max25_ (a and b) and *J*_max25_/*V*_*c*max25_ (c and d) after 1 and 8 days of acclimation (*T*_air_ = 15°C:25°C or 5°C:15°C) from warm to cool as well as cool to warm regimes, compared to control plants. Here, *V*_*c*max25_ and *J*_max25_ data reflect measurements corrected to a common *T*_leaf_ of 25°C. Different letters in panels indicate significant differences between combinations of different accessions, acclimation days and acclimation regimes (linear mixed models, *p* < 0.05). Between factor comparisons from the linear mixed models across the entire data set are summarised in the inset tables, with further details of the model results shown in the Supporting Information. [Color figure can be viewed at wileyonlinelibrary.com]

The photosynthetic variables derived from the relationships between *A*_sat_ and *C*_i_ for each plant are shown in Table S3. *V*_*c*max25_ differed significantly based on acclimation regime (*T*_air_ = 15°C:25°C or 5°C:15°C), but there was no significant difference in *V*_*c*max25_ between 1 and 8 days of acclimation (Figure 4, *p* = 0.753). *V*_*c*max_ also differed significantly based on acclimation regime but, again, there was no significant difference in *V*_*c*max_ between 1 and 8 days of acclimation (Figure S7, *p* = 0.419). One day after the switch, plants of both accessions that were either grown at 15°C:25°C or were acclimated to 15°C:25°C had a 1.5‐fold lower *V*_*c*max25_ than plants that were either grown at 5°C:15°C or were acclimated to 5°C:15°C. This difference also held 8 days after the switch.

The same generalised responses were evident for *J*_max25_ and *J*_max_ with a significant difference due to acclimation temperature, but no difference between acclimation day (Table S4, *p* = 0.638 and *p* = 0.273 for *J*_max25_ and *J*_max_, respectively). Thus, for both *V*_*c*max25_ and *V*_*c*max_ and *J*_max25_ and *J*_max_, differences between acclimation regimes were already evident by Day 1, and no further change was detected between Day 1 and Day 8 under our conditions. Note that, in theory, the different direction of change for *V*_*c*max_ and *J*_max_ versus *V*_*c*max25_ and *J*_max_25 reflects the effect of *T*_leaf_ on enzymatic kinetics for the former variables and the effect of *T*_leaf_ on biochemical capacity for the latter variables.

The ratio of *J*_max25_/*V*_*c*max25_ also differed significantly based on acclimation regime (*T*_air_ = 15°C:25°C or 5°C:15°C), but in this case, there was an interaction between acclimation day and acclimation temperature (Figure 4, *p* < 0.001). For plants grown at a *T*_air_ of 15°C:25°C, then transferred to a *T*_air_ of 5°C:15°C, *J*_max25_/*V*_*c*max25_ was lower after 1 day of acclimation compared to *J*_max25_/*V*_*c*max25_ measured on the same plants after 8 days of acclimation. Similarly, plants that were grown at a *T*_air_ of 5°C:15°C, then transferred to a *T*_air_ of 15°C:25°C had higher *J*_max25_/*V*_*c*max25_ after 1 day of acclimation compared to after 8 days of acclimation. The same interaction was evident for the ratio of *J*_max_/*V*_*c*max_ (Figure S7, *p* < 0.001).

From the relationships between *J*_max_ and *V*_*c*max_ and *J*_max25_ and *V*_*c*max25_, it was observed that the intercepts of the fitted models differed between plants that were grown at a *T*_air_ of 15°C:25°C versus plants that were acclimated for 1 day at 15°C:25°C (Figure S8a,c and Table S5, *p* < 0.001). The same difference in the intercepts of the fitted models was evident for plants that were grown at 5°C:15°C versus plants that were acclimated for 1 day at 5°C:15°C (Figure S8a,c and Table S5, *p* < 0.001). However, for these same between‐group comparisons, there were no differences in the intercepts of the fitted models after 8 days of temperature acclimation (Figure S8b,d and Table S5, *p* > 0.05). *V*_*c*max_ was significantly greater for accession CAF‐Warm compared to accession CAF‐Cool, but no other variable derived from the relationships between *A*_sat_ and *C*_i_ showed a significant between‐accession difference.

**Figure 5:**
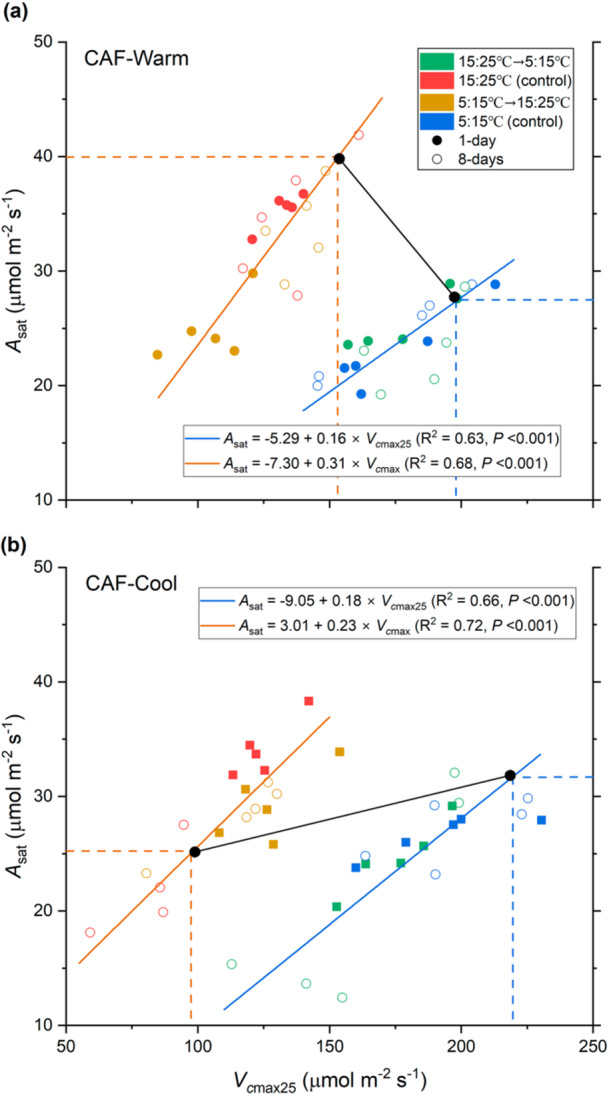
Relationships between *A*_sat_ and *V*_*c*max_ for accession CAF‐Warm (a) and CAF‐Cool (b). Here, *A*_sat_ data reflect non‐temperature‐corrected measurements conducted at 15°C and 25°C air temperature for plants growing in the L and H temperature regimes, respectively, and *V*_*c*max25_ data reflect measurements corrected to a common temperature of 25°C—a measure of carboxylation capacity without the influence of temperature on enzymatic kinetics. Fitted lines are SMA regression models with data aggregated into groups based on tests for differences between the slope and intercept of treatment combinations. The output of the tests is shown in the Supporting Information. The dashed orange and blue lines represent, respectively, the *V*_*c*max25_ predicted from the average *A*_sat_ measured from the near‐instantaneous responses at a *T*_leaf_ of 25°C and 15°C (*Experiment 1*). The solid black lines depict the change from warm‐grown to cool‐grown plants. [Color figure can be viewed at wileyonlinelibrary.com]

To understand the implications of changes in carboxylation capacity on photosynthesis owing to shifts in temperature regime, we examined the relationships between *A*_sat_ and *V*_*c*max25_. *A*_sat_ measured after 1 and 8 days of temperature acclimation was positively correlated with *V*_*c*max25_ (Figure 5 and Table S6). Repeated measures correlation results are shown in Table S7. From the correlations between *A*_sat_ and *V*_*c*max25_ (Figure 5a,b), a between‐treatment analysis of the slope and intercept revealed distinct subgroups. For both accessions, there were no differences in the slopes or intercepts of the linear models fit to the data of plants grown at 15°C:25°C or transferred to 15°C:25°C (*p* > 0.05). Similarly, for both accessions, there was no difference in the slope or intercept of the models fit to data from plants grown at 5°C:15°C or transferred to 5°C:15°C (*p* > 0.05). However, a comparison of the aggregate groups (plants grown or transferred to 5°C:15°C versus plants grown or transferred to 15°C:25°C) showed distinct differences. For accession CAF‐Warm, the slopes of the linear models fit to the data from these groups were significantly different (*p* = 0.002, Table S6 and Figure 5a). For CAF‐Cool, there was no difference in the slopes of the linear models fit to the data from these groups, but the intercepts of the linear models were significantly different (*p* < 0.001, Table S6 and Figure 5b). Overall, there was a tendency for a greater *A*_sat_ for a given *V*_*c*max25_ in plants that were either grown in, or transferred to, the 15°C:25°C temperature regime.

## 4 Discussion

### 4.1 Temperature Response Functions

The near‐instantaneous responses of ΦPSII and *A*_sat_ to *T*_leaf_ were well described by rectangular functions (Figure 2), with the greatest rate of (negative) change in ΦPSII and *A*_sat_ with *T*_leaf_ occurring at a *T*_leaf_ above the thermal optima (refer to Figure S3 for plots of the first derivative of the temperature responses). Both accessions functionally acclimated to their growth temperature by modifying *T*_opt [ΦPSII]_ and *T*_opt [*A*sat]_, with the optimal temperature for ΦPSII and *A*_sat_ increasing in plants grown in a warm environment (Hypothesis 1). However, there was also evidence of thermal adaptation of ΦPSII and *A*_sat_ (Hypothesis 1). ΦPSII_[max]_ and *A*_sat[max]_ were higher in warm‐grown plants of accession CAF‐Warm (developed in a warm climate), whereas ΦPSII_[max]_ and *A*_sat[max]_ were higher in cool‐grown plants of accession CAF‐Cool (developed in a cool climate). Although our comparison is limited to two accessions, this could mean that while the thermal optima for ΦPSII photochemistry and net photosynthesis acclimate to the prevailing temperature, the maximum capacity of these variables is influenced by genotypic adaptation.

For a given *T*_opt [*A*sat]_, *T*_opt [ΦPSII]_ was generally higher, suggesting that the thermal optimum of PSII ETR was consistently greater than for light‐saturated net photosynthesis (Figure 3a). This is not surprising given that net photosynthesis is gross photosynthesis (or true photosynthesis) minus photorespiration and dark respiration (Wohlfahrt and Gu 2015), with the temperature effect of photorespiration and dark respiration likely to reduce *T*_opt [*A*sat]_ more so than *T*_opt [ΦPSII]_. Furthermore, ΦPSII mainly reflects the photochemical efficiency of PSII in the chloroplasts near the leaf surface, whereas *A*_sat_ integrates whole‐leaf CO_2_ assimilation, including mesophyll diffusion and respiratory losses. These additional biochemical and diffusional constraints likely lower the temperature optimum of *A*_sat_ relative to ΦPSII. Notwithstanding these effects, there was a clear linear relationship between ΦPSII_[max]_ and *A*_sat[max]_ (Figure 3b). Thus, for the accessions and growth conditions of this study, the maximum attainable net photosynthetic rate was coordinated with the maximum attainable PSII ETR (Hypothesis 1). Note also that *J*_max_ and *J*_max25_ (discussed further in Section 4.2, below), which describe the potential electron transport for RuBP regeneration, are not mechanistically identical to PSII electron transport itself. Thus, while the coordination among ΦPSII, *A*_sat_, and *J*_max_ (*J*_max25_) reflects an integrated response of photochemistry and biochemistry, these variables do not necessarily respond to temperature with the same sensitivity or on the same timescale.

Cool‐grown plants of accession CAF‐Warm (warm adapted) showed a marked decline in ΦPSII and *A*_sat_ at 40°C (depicted as ΦPSII_[40]_ and *A*_sat[40]_, respectively) compared to warm‐grown plants of the same accession (Figure S6a,b). Thus, for the accession developed in a warm environment, it appears that the photosynthetic apparatus can be primed for operation at high temperatures when grown at modestly high temperatures. The same pattern was not evident for plants of accession CAF‐Cool (developed in a cool environment), which might mean that adaptation to cool conditions can be at the expense of a capacity to acclimate to high temperatures, although further research drawing from more accessions would be needed to confirm this possibility. The benefits of heat priming have been observed in several species (Fan et al. 2018; Zhou et al. 2020; Liu et al. 2021). Generally, heat priming leads to photosynthetic acclimation, quantified as a relative gain in photosynthetic efficiency, at high temperatures. In relation to ΦPSII_[40]_ and *A*_sat[40]_, the different responses of the two accessions of this study were consistent with genotypic thermal priming, whereby the progenies of primed plants exhibit upregulation of genes coding for heat‐tolerant enzymes and complexes of the photosynthetic apparatus (Wang et al. 2016). While there was evidence for genotypic thermal priming for accession CAF‐Warm (in relation to temperature increases), the process appears to be reduced or absent for accession CAF‐Cool. Such differential thermal responses have been observed across species. For example, Smith and Dukes (2017) noted the effect across plant types, and Wittemann et al. (2022) observed a similar pattern across a thermal gradient. However, we are unaware of published results showing distinct between‐accession differences.

Unlike ΦPSII and *A*_sat_ at 40°C, the relative change in ΦPSII and *A*_sat_ from the maximum value to the value recorded at 40°C (ΔΦPSII and Δ*A*_sat_, respectively) was responsive to growth temperature for plants of both accessions (Figure S6c,d). For both accessions, ΔΦPSII and Δ*A*_sat_ were greater (more negative) for cool‐grown plants than for warm‐grown plants. Greater downregulation of the enzymes and structures responsible for photosynthesis in cool‐grown plants thus appears to be conserved regardless of the climate under which an accession was developed. This points to the idea that partial thermal acclimation is a universal feature of photosynthesis (Kumarathunge et al. 2019; Tarvainen et al. 2022), but the temperature response of specific elements of the photosynthetic apparatus may depend on the climate under which a plant has evolved.

### 4.2 Timeframes for Acclimation

When assessing the timeframes for photosynthetic acclimation, we focus on the effect of temperature on biochemical and photochemical capacity, quantified as *V*_*c*max25_ and *J*_max25_, respectively. The *V*_*c*max25_ and *J*_max25_ of both *B. oleracea* accessions acclimated to the temperature regimes experienced during plant growth and the temperature regimes experienced following the transfer step, termed long‐term and short‐term acclimation, respectively, from hereon (Figure 4). Long‐term and short‐term acclimation to a cool environment resulted in a higher *V*_*c*max25_ and *J*_max25_ than long‐term and short‐term acclimation to a warm environment.

The similar acclimation responses of *V*_*c*max25_ and *J*_max25_ to temperature underscores the commonly observed covariation of *V*_*c*max25_ and *J*_max25_ (Wullschleger 1993; Kattge and Knorr 2007; Walker et al. 2014), although subtle differences in the acclimation timeframes of these variables were observed (see further discussion on this point below). Notwithstanding the between‐treatment effects, there were no discernible differences in the response of *V*_*c*max25_ and *J*_max25_ to temperature between accessions, suggesting little evidence for thermal adaptation of these variables in these accessions. However, across species, we note that Rubisco kinetic parameters appear to adapt to thermal regimes (Galmés et al. 2016).

Further empirical evidence for a generalised requirement for a greater *V*_*c*max25_ to support a greater rate of net photosynthesis has been shown by Scafaro et al. (2017), drawing from a selection of tree species that were grown at different temperatures. Scafaro et al. (2017) also demonstrated that the observed acclimation of *V*_*c*max25_ to temperature required an adjustment to the amount of Rubisco in leaves and associated changes in the allocation of N to this enzyme. However, in our study, the timeframe for the short‐term acclimation of *V*_*c*max25_ to temperature was < 1 day (contrary to Hypothesis 2)—there was no difference in *V*_*c*max25_ between 1 and 8 days of temperature acclimation. This timeframe conflicts with our hypothesis and the assumption that the production of different amounts or forms of Rubisco are required for carboxylation capacity to undergo thermal acclimation, given that the half‐life of Rubisco is approximately 1 week (Simpson et al. 1981). While the exact nature of the fast Rubisco acclimation response of our study is unclear, it is possible that forms of the enzymatic chaperone, Rubisco activase, responded to the short‐term acclimation temperature of our experiment (Law and Crafts‐Brandner 2001; Yamori et al. 2014). Alternatively, rapid acclimation to warm temperatures may have involved Rubisco deactivation, a process that Scafaro et al. (2023) incorporated into a model to account for decreased net photosynthesis at elevated temperatures. Regardless of the mechanism, a rapid acclimation response for carboxylation capacity would be a competitive advantage for plants with a relatively short growing season or a high leaf turnover rate, where there is an imperative to maximise productivity in a dynamic temperature environment over a relatively short period (such as the plants used in this study). Indeed, it is possible that the acclimation timeframe for *V*_*c*max_ and *V*_*c*max25_ varies across plant functional groups and may map onto other traits, such as those of the leaf economics spectrum (Wright et al. 2004).

The *J*_max25_ to *V*_*c*max25_ ratio (*J*_max25_/*V*_*c*max25_) also responded to both long‐term and short‐term temperature acclimation. For plants of both accessions, *J*_max25_/*V*_*c*max25_ decreased with long‐term and short‐term acclimation to cool temperatures and increased with long‐term and short‐term acclimation to warm temperatures (Figure 4c,d). These results are consistent with the meta‐analysis of Kattge and Knorr (2007) and the experimental observation for C_3_ species of Smith and Dukes (2017). Thus overall, and as noted for other C_3_ species, rates of *V*_*c*max25_ were enhanced relative to rates of *J*_max25_ with acclimation to cooler temperatures in the *B. oleracea* accessions of this study. However, unlike the trends observed for each of these variables separately, the timeframe for the thermal acclimation of *J*_max25_/*V*_*c*max25_ and was more nuanced. For both accessions and acclimation temperatures, *J*_max25_/*V*_*c*max25_ differed between 1‐day and 8‐days of short‐term acclimation. The reason for this difference can be observed by comparing the relative change of *V*_*c*max25_ and *J*_max25_ between 1‐day and 8‐days of temperature acclimation (see Figure S8 and Table S5). For a given *V*_*c*max25_, *J*_max25_ was slightly higher for the plants acclimated to 1 day of cool temperatures compared to control plants that were grown under cool temperatures. Similarly, for a given *V*_*c*max25_, *J*_max25_ was slightly lower for the plants acclimated to 1 day of warm temperatures compared to control plants that were grown under warm conditions. After 8 days of short‐term acclimation, these differences were not evident. Thus, there appears to a slight lag between the acclimation response of *J*_max25_ compared to *V*_*c*max25_. This suggests that, over this short time period, thermal acclimation of the electron transport component of photosynthesis is slightly delayed compared to the acclimation of Rubisco, which would mean that a coordinated photosynthetic response to temperature shifts may be slightly longer than the response of carboxylation in isolation. The reason for the delayed response of *J*_max25_ could relate to the need for structural rearrangement of components of the electron transport chain, as observed for acclimation to light (Walters 2005), a process that could take longer than altering the forms or quantities of Rubisco or Rubisco activase.

### 4.3 Implications for Photosynthesis

The effects of the thermal acclimation responses of *V*_*c*max25_ on photosynthesis can be examined from the relationships between these variables and *A*_sat_ (Figure 5), noting that *V*_*c*max25_ represents the maximum temperature‐standardised carboxylation capacity, reflecting biochemical investment in Rubisco. For both accessions, clear linear relationships between *A*_sat_ and *V*_*c*max25_ were evident, with data grouped by long‐term and short‐term acclimation temperatures. Thus, for each group, a greater *A*_sat_ was supported by a greater investment in *V*_*c*max25_, which is consistent with observations and theory (Kattge et al. 2009; Maire et al. 2012). However, for a given *V*_*c*max25_, *A*_sat_ was greater for plants that were either grown in, or transferred to, a warm environment; that is, the intercept of the relationship between *A*_sat_ and *V*_*c*max25_ for these plants was greater than for plants that were either grown in, or transferred to, a cool environment. Thus, growth or short‐term acclimation to a warm environment results in de‐investment in carboxylation capacity. However, for a given *V*_*c*max25_, *A*_sat_ is enhanced under these same conditions because of the effect of elevated temperatures on enzymatic kinetics. These responses were evident after only 1 day of thermal acclimation.

Note that, due to practical limitations in regulating RH, the VPD of the growth rooms did differ. Future studies may aim to address this issue by selecting a smaller difference in *T*_air_ between growth conditions, thereby enabling manipulation of RH to achieve the same VPD.

### 4.4 Synthesis of Experimental Observations

Referring to the results of *Experiment 2*, the dashed lines in Figure 5a,b represent the average *A*_sat_ measured from the near‐instantaneous responses of *Experiment 1* at a *T*_leaf_ of 15°C for plants grown at 5°C:15°C and a *T*_leaf_ of 25°C for plants grown at 15°C:25°C, that is, a *T*_leaf_ equivalent to the maximum *T*_air_ of the daytime photoperiod of each group of plants. The dashed lines are extended to the associated *V*_*c*max25_ values predicted by the linear models: 15°C measurements extend to the 5°C:15°C *A*_sat_∼*V*_*c*max_ linear model, 25°C measurements extend to the 15°C:25°C *A*_sat_∼*V*_*c*max_ linear model. For simplicity, the linear models used for this representation are aggregations of plants that were grown and acclimated to either 15°C:25°C or 5°C:15°C.

The predicted values of *V*_*c*max25_ from this exercise would be equivalent to the H (control) and L (control) values of Figure 4a,b, respectively. From Figure 5, it can be seen that a higher *V*_*c*max25_ would be predicted for plants of both accessions that were grown at 5°C:15°C and measured at a *T*_leaf_ of 15°C, even though *A*_sat_ was higher for CAF‐Warm plants grown at 15°C:25°C and measured at 25°C. Referring to Figure 4a,b, it can be seen that *V*_*c*max25_ is lower for H (control) plants compared to L (control) plants of both accessions, which is consistent with this prediction. In other words, the observed between‐accession difference in *A*_sat[max]_ (*Experiment 1*) could occur without a between‐accession difference in the *V*_*c*max25_ temperature response (*Experiment 2*). Overall, there was a generalised requirement for a greater *V*_*c*max25_ in cool‐grown plants of both accessions in order to compensate for reduced enzymatic kinetics at these lower temperatures (Togashi et al. 2018).

### 4.5 Implications for Modelling

We have shown a clear response of ΦPSII to temperature, with evidence of functional acclimation and adaptation, albeit from two accessions. At the leaf scale, models such as the FvCB model of C_3_ photosynthesis typically do not consider a temperature response function for ΦPSII. Adding a ΦPSII temperature response function to leaf photosynthesis models may improve the temperature sensitivity of RuBP regeneration‐limited photosynthesis, which may be especially important when estimating photosynthesis above and below the thermal optima.

We have also demonstrated that thermal acclimation of the maximum rate of carboxylation can occur within 1 day of exposure to a new temperature regime. This rapid response has implications for crop production or land surface carbon cycling models that aim to make predictions on short‐ to medium‐term timescales. For example, given the strong relationship between *A*_sat_ and *V*_*c*max25_, it is likely that a rapid acclimation of the maximum rate of carboxylation at the scale of a canopy will also rapidly alter the gross primary productivity.

## Conflicts of Interest

The authors declare no conflicts of interest.

## Supporting information

Supporting File.

## Data Availability

The data underlying each figure are available in the following perpetual data repository: https://research-repository.uwa.edu.au/.

## References

[pce70555-bib-0001] Bakdash, J. Z. , and L. R. Marusich . 2017. “Repeated Measures Correlation.” Frontiers in Psychology 8. https://www.frontiersin.org/journals/psychology/articles/10.3389/fpsyg.2017.00456/full. 10.3389/fpsyg.2017.00456 PMC5383908 28439244

[pce70555-bib-0002] Bernacchi, C. J. , E. L. Singsaas , C. Pimentel , A. R. Portis, Jr. , and S. P. Long . 2001. “Improved Temperature Response Functions for Models of Rubisco‐Limited Photosynthesis.” Plant, Cell & Environment 24: 253–259.

[pce70555-bib-0003] Berry, J. , and O. Bjorkman . 1980. “Photosynthetic Response and Adaptation to Temperature in Higher Plants.” Annual Review of Plant Physiology 31: 491–543.

[pce70555-bib-0004] Box, G. E. P. , and D. R. Cox . 1964. “An Analysis of Transformations.” Journal of the Royal Statistical Society Series B: Statistical Methodology 26: 211–243.

[pce70555-bib-0005] Brooks, A. , and G. D. Farquhar . 1985. “Effect of Temperature on the Co_2_/O_2_ Specificity of Ribulose‐1,5‐bisphosphate Carboxylase Oxygenase and the Rate of Respiration in the Light ‐ Estimates From Gas‐Exchange Measurements on Spinach.” Planta 165: 397–406. 24241146 10.1007/BF00392238

[pce70555-bib-0006] Campbell, C. , L. Atkinson , J. Zaragoza‐Castells , M. Lundmark , O. Atkin , and V. Hurry . 2007. “Acclimation of Photosynthesis and Respiration Is Asynchronous in Response to Changes in Temperature Regardless of Plant Functional Group.” New Phytologist 176: 375–389. 17692077 10.1111/j.1469-8137.2007.02183.x

[pce70555-bib-0007] Collatz, G. J. , J. T. Ball , C. Grivet , and J. A. Berry . 1991. “Physiological and Environmental‐Regulation of Stomatal Conductance, Photosynthesis and Transpiration ‐ A Model That Includes a Laminar Boundary‐Layer.” Agricultural and Forest Meteorology 54: 107–136.

[pce70555-bib-0008] Coumou, D. , and A. Robinson . 2013. “Historic and Future Increase in the Global Land Area Affected by Monthly Heat Extremes.” Environmental Research Letters 8: 034018.

[pce70555-bib-0009] Dusenge, M. E. , A. G. Duarte , and D. A. Way . 2019. “Plant Carbon Metabolism and Climate Change: Elevated CO_2_ and Temperature Impacts on Photosynthesis, Photorespiration and Respiration.” New Phytologist 221: 32–49. 29983005 10.1111/nph.15283

[pce70555-bib-0010] Duursma, R. A. 2015. “Plantecophys ‐ An R Package for Analysing and Modelling Leaf Gas Exchange Data.” PLoS One 10: e0143346. 26581080 10.1371/journal.pone.0143346 PMC4651500

[pce70555-bib-0011] Fan, Y. , C. Ma , Z. Huang , et al. 2018. “Heat Priming During Early Reproductive Stages Enhances Thermo‐Tolerance to Post‐Anthesis Heat Stress via Improving Photosynthesis and Plant Productivity in Winter Wheat (*Triticum aestivum* L.).” Frontiers in Plant Science 9: 17. 29951079 10.3389/fpls.2018.00805 PMC6008404

[pce70555-bib-0012] Farquhar, G. D. , S. von Caemmerer , and J. A. Berry . 1980. “A Biochemical‐Model of Photosynthetic CO_2_ Assimilation in Leaves of C‐3 Species.” Planta 149: 78–90. 24306196 10.1007/BF00386231

[pce70555-bib-0013] Feller, U. , S. J. Crafts‐Brandner , and M. E. Salvucci . 1998. “Moderately High Temperatures Inhibit Ribulose‐1,5‐Bisphosphate Carboxylase/Oxygenase (Rubisco) Activase‐Mediated Activation of Rubisco.” Plant Physiology 116: 539–546. 9490757 10.1104/pp.116.2.539 PMC35111

[pce70555-bib-0014] Galmés, J. , C. Hermida‐Carrera , L. Laanisto , and Ü. Niinemets . 2016. “A Compendium of Temperature Responses of Rubisco Kinetic Traits: Variability Among and Within Photosynthetic Groups and Impacts on Photosynthesis Modeling.” Journal of Experimental Botany 67: 5067–5091. 27406782 10.1093/jxb/erw267 PMC5014154

[pce70555-bib-0015] Garen, J. C. , H. A. Branch , I. Borrego , B. Blonder , J. R. Stinziano , and S. T. Michaletz . 2022. “Gas Exchange Analysers Exhibit Large Measurement Error Driven by Internal Thermal Gradients.” New Phytologist 236: 369–384. 35762843 10.1111/nph.18347

[pce70555-bib-0016] Genty, B. , J. M. Briantais , and N. R. Baker . 1989. “The Relationship Between the Quantum Yield of Photosynthetic Electron‐Transport and Quenching of Chlorophyll Fluorescence.” Biochimica et Biophysica Acta (BBA) ‐ General Subjects 990: 87–92.

[pce70555-bib-0017] Haldimann, P. , and U. Feller . 2004. “Inhibition of Photosynthesis by High Temperature in Oak (*Quercus pubescens* L.) Leaves Grown Under Natural Conditions Closely Correlates With a Reversible Heat‐Dependent Reduction of the Activation State of Ribulose‐1,5‐bisphosphate Carboxylase/Oxygenase.” Plant, Cell & Environment 27: 1169–1183.

[pce70555-bib-0018] Hall, N. P. , and A. J. Keys . 1983. “Temperature‐Dependence of the Enzymic Carboxylation and Oxygenation of Ribulose 1,5‐bisphosphate in Relation to Effects of Temperature on Photosynthesis.” Plant Physiology 72: 945–948. 16663143 10.1104/pp.72.4.945 PMC1066354

[pce70555-bib-0019] Havaux, M. 1992. “Stress Tolerance of Photosystem‐II In Vivo — Antagonistic Effects of Water, Heat, and Photoinhibition Stresses.” Plant Physiology 100: 424–432. 16652979 10.1104/pp.100.1.424 PMC1075568

[pce70555-bib-0020] Havaux, M. 1993. “Rapid Photosynthetic Adaptation to Heat‐Stress Triggered in Potato Leaves by Moderately Elevated‐Temperatures.” Plant, Cell & Environment 16: 461–467.

[pce70555-bib-0021] IPCC . 2022. *Global Warming of 1.5°C: IPCC Special Report on Impacts of Global Warming of 1.5°C Above Pre‐Industrial Levels in Context of Strengthening Response to Climate Change, Sustainable Development, and Efforts to Eradicate Poverty*. Cambridge University Press.

[pce70555-bib-0022] Kattge, J. , and W. Knorr . 2007. “Temperature Acclimation in a Biochemical Model of Photosynthesis: A Reanalysis of Data From 36 Species.” Plant, Cell & Environment 30: 1176–1190. 10.1111/j.1365-3040.2007.01690.x 17661754

[pce70555-bib-0023] Kattge, J. , W. Knorr , T. Raddatz , and C. Wirth . 2009. “Quantifying Photosynthetic Capacity and Its Relationship to Leaf Nitrogen Content for Global‐Scale Terrestrial Biosphere Models.” Global Change Biology 15: 976–991.

[pce70555-bib-0024] Kumarathunge, D. P. , B. E. Medlyn , J. E. Drake , et al. 2019. “Acclimation and Adaptation Components of the Temperature Dependence of Plant Photosynthesis at the Global Scale.” New Phytologist 222: 768–784. 30597597 10.1111/nph.15668

[pce70555-bib-0025] Law, R. D. , and S. J. Crafts‐Brandner . 2001. “High Temperature Stress Increases the Expression of Wheat Leaf Ribulose‐1,5‐bisphosphate Carboxylase/Oxygenase Activase Protein.” Archives of Biochemistry and Biophysics 386: 261–267. 11368350 10.1006/abbi.2000.2225

[pce70555-bib-0026] Levenberg, K. 1944. “A Method for the Solution of Certain Non‐Linear Problems in Least Squares.” Quarterly of Applied Mathematics 2: 164–168.

[pce70555-bib-0027] Lindstrom, M. J. , and D. M. Bates . 1990. “Nonlinear Mixed Effects Models for Repeated Measures Data.” Biometrics 46: 673–687. 2242409

[pce70555-bib-0028] Liu, B. , L. Zhang , L. Rusalepp , et al. 2021. “Heat Priming Improved Heat Tolerance of Photosynthesis, Enhanced Terpenoid and Benzenoid Emission and Phenolics Accumulation in *Achillea millefolium* .” Plant, Cell & Environment 44: 2365–2385. 10.1111/pce.13830 32583881

[pce70555-bib-0029] Maire, V. , P. Martre , J. Kattge , et al. 2012. “The Coordination of Leaf Photosynthesis Links C and N Fluxes in C_3_ Plant Species.” PLoS One 7: e38345. 22685562 10.1371/journal.pone.0038345 PMC3369925

[pce70555-bib-0030] Marquardt, D. W. 1963. “An Algorithm for Least‐Squares Estimation of Nonlinear Parameters.” Journal of the Society for Industrial and Applied Mathematics 11: 431–441.

[pce70555-bib-0031] Mawson, B. T. , and W. R. Cummins . 1989. “Thermal‐Acclimation of Photosynthetic Electron‐Transport Activity by Thylakoids of *Saxifraga‐cernua* .” Plant Physiology 89: 325–332. 16666534 10.1104/pp.89.1.325 PMC1055839

[pce70555-bib-0032] Maxwell, K. , and G. N. Johnson . 2000. “Chlorophyll Fluorescence — A Practical Guide.” Journal of Experimental Botany 51: 659–668. 10938857 10.1093/jxb/51.345.659

[pce70555-bib-0033] Medlyn, B. E. , E. Dreyer , D. Ellsworth , et al. 2002. “Temperature Response of Parameters of a Biochemically Based Model of Photosynthesis. II. A Review of Experimental Data.” Plant, Cell & Environment 25: 1167–1179.

[pce70555-bib-0034] Mitchell, R. A. C. , and J. Barber . 1986. “Adaptation of Photosynthetic Electron‐Transport Rate to Growth Temperature in Pea.” Planta 169: 429–436. 24232657 10.1007/BF00392141

[pce70555-bib-0035] Miyazawa, Y. , and K. Kikuzawa . 2006. “Physiological Basis of Seasonal Trend in Leaf Photosynthesis of Five Evergreen Broad‐Leaved Species in a Temperate Deciduous Forest.” Tree Physiology 26: 249–256. 16356922 10.1093/treephys/26.2.249

[pce70555-bib-0036] Murchie, E. H. , and T. Lawson . 2013. “Chlorophyll Fluorescence Analysis: A Guide to Good Practice and Understanding Some New Applications.” Journal of Experimental Botany 64: 3983–3998. 23913954 10.1093/jxb/ert208

[pce70555-bib-0037] Neri, P. , L. Gu , and Y. Song . 2024. “The Effect of Temperature on Photosystem II Efficiency Across Plant Functional Types and Climate.” Biogeosciences 21: 2731–2758.

[pce70555-bib-0038] NOAA . 2024. Global Historical Climatology Network. NOAA NCEI. 10.7289/V5222RT1. PMC11180175 38879587

[pce70555-bib-0039] Oberhuber, W. , and G. E. Edwards . 1993. “Temperature‐Dependence of the Linkage of Quantum Yield of Photosystem‐II to CO_2_ Fixation in C‐4 and C‐3 Plants.” Plant Physiology 101: 507–512. 12231705 10.1104/pp.101.2.507 PMC160598

[pce70555-bib-0040] Pinheiro, J. , and D. M. Bates , and R Core Team . 2026. “nlme: Linear and Nonlinear Mixed Effects Models.” https://CRAN.R-project.org/package=nlme.

[pce70555-bib-0041] Porcar‐Castell, A. 2011. “A High‐Resolution Portrait of the Annual Dynamics of Photochemical and Non‐Photochemical Quenching in Needles of *Pinus sylvestris* .” Physiologia Plantarum 143: 139–153. 21615415 10.1111/j.1399-3054.2011.01488.x

[pce70555-bib-0042] R Development Core Team . 2025. *R: A Language and Environment for Statistical Computing*. R Foundation for Statistical Computing. https://www.R-project.org/.

[pce70555-bib-0043] Ren, Y. , H. Wang , S. P. Harrison , et al. 2024. “Reduced Global Plant Respiration Due to the Acclimation of Leaf Dark Respiration Coupled With Photosynthesis.” New Phytologist 241: 578–591. 37897087 10.1111/nph.19355

[pce70555-bib-0044] Rogers, A. , B. E. Medlyn , J. S. Dukes , et al. 2017. “A Roadmap for Improving the Representation of Photosynthesis in Earth System Models.” New Phytologist 213: 22–42. 27891647 10.1111/nph.14283

[pce70555-bib-0045] Sage, R. F. , and D. S. Kubien . 2007. “The Temperature Response of C_3_ and C_4_ Photosynthesis.” Plant, Cell & Environment 30: 1086–1106. 10.1111/j.1365-3040.2007.01682.x 17661749

[pce70555-bib-0046] Salisbury, F. B. , and C. W. Ross . 1985. Plant Physiology. Wadsworth Inc.

[pce70555-bib-0047] Scafaro, A. P. , B. C. Posch , J. R. Evans , G. D. Farquhar , and O. K. Atkin . 2023. “Rubisco Deactivation and Chloroplast Electron Transport Rates Co‐Limit Photosynthesis Above Optimal Leaf Temperature in Terrestrial Plants.” Nature Communications 14: 2820. 10.1038/s41467-023-38496-4 PMC10192301 37198175

[pce70555-bib-0048] Scafaro, A. P. , S. Xiang , B. M. Long , et al. 2017. “Strong Thermal Acclimation of Photosynthesis in Tropical and Temperate Wet‐Forest Tree Species: The Importance of Altered Rubisco Content.” Global Change Biology 23: 2783–2800. 27859952 10.1111/gcb.13566

[pce70555-bib-0049] Schreiber, U. , and J. A. Berry . 1977. “Heat‐Induced Changes of Chlorophyll Fluorescence in Intact Leaves Correlated With Damage of the Photosynthetic Apparatus.” Planta 136: 233–238. 24420396 10.1007/BF00385990

[pce70555-bib-0050] Shapiro, S. S. , and M. B. Wilk . 1965. “An Analysis of Variance Test for Normality (Complete Samples).” Biometrika 52: 591–611.

[pce70555-bib-0051] Simpson, E. , R. J. Cooke , and D. D. Davies . 1981. “Measurement of Protein Degradation in Leaves of *Zea mays* Using [3H]Acetic Anhydride and Tritiated Water.” Plant Physiology 67: 1214–1219. 16661839 10.1104/pp.67.6.1214 PMC425864

[pce70555-bib-0052] Smith, N. G. , and J. S. Dukes . 2017. “Short‐Term Acclimation to Warmer Temperatures Accelerates Leaf Carbon Exchange Processes Across Plant Types.” Global Change Biology 23: 4840–4853. 28560841 10.1111/gcb.13735

[pce70555-bib-0053] Tarvainen, L. , M. Wittemann , M. Mujawamariya , et al. 2022. “Handling the Heat ‐ Photosynthetic Thermal Stress in Tropical Trees.” New Phytologist 233: 236–250. 34655491 10.1111/nph.17809

[pce70555-bib-0054] Togashi, H. , I. C. Prentice , O. K. Atkin , et al. 2018. “Thermal Acclimation of Leaf Photosynthetic Traits in an Evergreen Woodland, Consistent With the Coordination Hypothesis.” Biogeosciences 15: 3461–3474.

[pce70555-bib-0055] van der Tol, C. , J. A. Berry , P. K. E. Campbell , and U. Rascher . 2014. “Models of Fluorescence and Photosynthesis for Interpreting Measurements of Solar‐Induced Chlorophyll Fluorescence.” Journal of Geophysical Research: Biogeosciences 119: 2312–2327. 27398266 10.1002/2014JG002713 PMC4852699

[pce70555-bib-0056] Vico, G. , D. A. Way , V. Hurry , and S. Manzoni . 2019. “Can Leaf Net Photosynthesis Acclimate to Rising and More Variable Temperatures?” Plant, Cell & Environment 42: 1913–1928. 10.1111/pce.13525 30706948

[pce70555-bib-0057] Viovy, N. , P. Ciais , A. Bastos , F. Maignan , C. Bacour , and M. Peaucelle . Forthcoming. “Heat Stress: An Underestimated Impact of Climate Change on Vegetation.” *Nature Portfolio*. Preprint. https://www.researchsquare.com/article/rs-4964815/v1.

[pce70555-bib-0058] Walker, A. P. , A. P. Beckerman , L. Gu , et al. 2014. “The Relationship of Leaf Photosynthetic Traits – *V* _cmax_ and *J* _max_ – to Leaf Nitrogen, Leaf Phosphorus, and Specific Leaf Area: A Meta‐Analysis and Modeling Study.” Ecology and Evolution 4: 3218–3235. 25473475 10.1002/ece3.1173 PMC4222209

[pce70555-bib-0059] Walters, R. G. 2005. “Towards an Understanding of Photosynthetic Acclimation.” Journal of Experimental Botany 56: 435–447. 15642715 10.1093/jxb/eri060

[pce70555-bib-0060] Wang, X. , C. Xin , J. Cai , et al. 2016. “Heat Priming Induces *trans*‐Generational Tolerance to High Temperature Stress in Wheat.” Frontiers in Plant Science 7: 12. 27148324 10.3389/fpls.2016.00501 PMC4830833

[pce70555-bib-0061] Warton, D. I. , R. A. Duursma , D. S. Falster , and S. Taskinen . 2012. “smatr 3‐an R Package for Estimation and Inference About Allometric Lines.” Methods in Ecology and Evolution 3: 257–259.

[pce70555-bib-0062] Wittemann, M. , M. X. Andersson , B. Ntirugulirwa , L. Tarvainen , G. Wallin , and J. Uddling . 2022. “Temperature Acclimation of Net Photosynthesis and Its Underlying Component Processes in Four Tropical Tree Species.” Tree Physiology 42: 1188–1202. 35038330 10.1093/treephys/tpac002 PMC9190752

[pce70555-bib-0063] Wohlfahrt, G. , and L. Gu . 2015. “The Many Meanings of Gross Photosynthesis and Their Implication for Photosynthesis Research From Leaf to Globe.” Plant, Cell & Environment 38: 2500–2507. 10.1111/pce.12569 PMC4681079 25988305

[pce70555-bib-0064] Wright, I. J. , P. B. Reich , M. Westoby , et al. 2004. “The Worldwide Leaf Economics Spectrum.” Nature 428: 821–827. 15103368 10.1038/nature02403

[pce70555-bib-0065] Wullschleger, S. D. 1993. “Biochemical Limitations to Carbon Assimilation in C_3_ Plants – A Retrospective Analysis of the *A*/*C_i_ * Curves From 109 Species.” Journal of Experimental Botany 44: 907–920.

[pce70555-bib-0066] Yamori, W. , K. Hikosaka , and D. A. Way . 2014. “Temperature Response of Photosynthesis in C_3_, C_4_, and CAM Plants: Temperature Acclimation and Temperature Adaptation.” Photosynthesis Research 119: 101–117. 23801171 10.1007/s11120-013-9874-6

[pce70555-bib-0067] Yamori, W. , K. Noguchi , Y. Kashino , and I. Terashima . 2008. “The Role of Electron Transport in Determining the Temperature Dependence of the Photosynthetic Rate in Spinach Leaves Grown at Contrasting Temperatures.” Plant and Cell Physiology 49: 583–591. 18296450 10.1093/pcp/pcn030

[pce70555-bib-0068] Yamori, W. , K. Suzuki , K. Noguchi , M. Nakai , and I. Terashima . 2006. “Effects of Rubisco Kinetics and Rubisco Activation State on the Temperature Dependence of the Photosynthetic Rate in Spinach Leaves From Contrasting Growth Temperatures.” Plant, Cell & Environment 29: 1659–1670. 10.1111/j.1365-3040.2006.01550.x 16898026

[pce70555-bib-0069] Yeo, I. K. 2000. “A New Family of Power Transformations to Improve Normality or Symmetry.” Biometrika 87: 954–959.

[pce70555-bib-0070] Zhou, R. , X. Yu , X. Li , T. Mendanha dos Santos , E. Rosenqvist , and C. O. Ottosen . 2020. “Combined High Light and Heat Stress Induced Complex Response in Tomato With Better Leaf Cooling After Heat Priming.” Plant Physiology and Biochemistry 151: 1–9. 32179467 10.1016/j.plaphy.2020.03.011

[pce70555-bib-0071] Zhu, L. , A. P. Scafaro , E. Vierling , et al. 2024. “Heat Tolerance of a Tropical‐Subtropical Rainforest Tree Species *Polyscias elegans*: Time‐Dependent Dynamic Responses of Physiological Thermostability and Biochemistry.” New Phytologist 241: 715–731. 37932881 10.1111/nph.19356

