# Supplementary material for: Temperature Acclimation of Chlorophyll Fluorescence and Carboxylation Capacity From Near‐Instantaneous to Weekly Time Scales
Source: Plant Cell Environ. 2026 Apr 22;49(8):5497–510. doi: 10.1111/pce.70555 (PMC13353689; doi:10.1111/pce.70555)
Supplement: Supplementary file 1 — Supporting File. [file PCE-49-5497-s001.docx]

**Supporting Information**

Plant, Cell and Environment Supporting Information: Temperature acclimation of chlorophyll fluorescence and carboxylation capacity from near-instantaneous to weekly time scales, Paul L. Drake, Erik J. Veneklaas, Nicholas G. Smith, Steven M. Driever and Hugo J. de Boer

The following Supporting Information is available for this article:

**Fig. S1** The range in climatic variables during the experiment for the 15:25℃ growth room, (a), (c) and (d), and the 5:15℃ growth room, (b), (d) and (f). *T*_air_: air temperature, RH: relative humidity, PAR: photosynthetically active radiation.

**Fig. S2:** Representation of the parameters derived from the response functions of ΦPSII and *A*_sat_ to *T*_leaf_. Parameters comprise: the maximum ΦPSII (ΦPSII_[max]_) and maximum *A*_sat_ (*A*_sat[max]_) over the range of measured *T*_leaf_, the *T*_leaf_ at ΦPSII_[max]_ and *A*_sat[max]_ (*T*_opt[ΦPSII]_ and *T*_opt[_*_A_*_sat]_, respectively), the ΦPSII and *A*_sat_ at a fixed *T*_leaf_ of 40℃ (ΦPSII_[40]_ and *A*_sat[40]_ for ΦPSII and *A*_sat_, respectively) and the relative difference between ΦPSII_[max]_ and ΦPSII_[40]_ (ΔΦPSII) and the relative difference between *A*_sat[max]_ and *A*_sat[40]_ (Δ*A*_sat_).

**Fig. S3** First derivatives of the temperature response functions of ΦPSII and *A*_sat_ in relation to *T*_leaf_. Thick lines are the average responses of all plants per temperature regime, thin lines are the individual responses of each plant.

**Fig. S4** Response of *g*_sw_ to *T*_leaf_ for each accession and growth temperature.

**Fig. S5** *T*_opt[ΦPSII]_ **(a)**, *T*_opt[_*_A_*_sat]_, **(b)**, ΦPSII_[max]_ **(c)** and *A*_sat[max]_ **(d)** in relation to the growth temperature for each accession. Different letters indicate significant differences between accessions and growth temperatures (Post hoc Tukey’s HSD tests, *P* < 0.05). Refer to Fig **S1** for a definition of the variables.

**Fig. S6** ΦPSII and *A*_sat_ recorded at a *T*_leaf_ of 40℃ (ΦPSII_[40]_ **(a)** and *A*_sat[40]_ **(b)**, respectively) and the change in ΦPSII and *A*_sat_ between ΦPSII_[max]_ and *A*_sat[max]_ (ΔΦPSII **(c)** and Δ*A*_sat_ **(d)**, respectively) in relation to the growth temperature (15:25℃ and 5:15℃) for each accession. Different letters indicate significant differences between accessions and growth temperatures (Post hoc Tukey’s HSD tests, *P* < 0.05). Refer to Fig. **S1** for a definition of the variables.

**Fig. S7** *V_c_*_max_ **(a, b)** and *J*_max_/*V_c_*_max_ **(c, d)** of each accession after 1-day and 8-days of temperature acclimation compared to control plants. The *V_c_*_max_ and *J*_max_ data reflect measurements at the daytime air temperature (15℃ or 25℃). Different letters in panels indicate significant differences between combinations of different accessions, acclimation days and acclimation temperatures (linear mixed models, *P* < 0.05). Between factor comparisons across the entire dataset from the linear mixed models are summarised in the inset tables, with further details of the model results shown in the Table **S3**.

**Fig. S8** Relationships between *J*_max_ and *V_c_*_max_ after 1-day of temperature acclimation **(a)** and 8-days of temperature acclimation **(b)**, and relationships between *J*_max_25 and *V_c_*_max_25 after 1-day **(c)** and 8-days **(d)** of temperature acclimation. Fitted lines are SMA regression models. Model comparisons are shown in Table **S4**.

**Table S1** Fitted parameters for the temperature response functions of ΦPSII and *A*_sat_.

Table **S2** Linear mixed effect model results for the near-instantaneous response of *g*_sw_ to *T*_leaf_. Df: degrees to freedom.

**Table S3** Photosynthetic variables derived from relationships between *A*_sat_ and *C*_i_. Switch refers to the number of days following the switch to the acclimation temperature.

**Table S4** Linear mixed effect model results for *V_c_*_max_, *V_c_*_max_25, *J*_max_, *J*_max_25, *J*_max_/*V_c_*_max_ and *J*_max_25/*V_c_*_max_25. df: degrees of freedom.

**Table S5** SMA test results for differences in the slope and intercept of the relationships between *J*_max_ and *V_c_*_max_ and *J*_max_25 and *V_c_*_max_25 after 1-day and 8-days of temperature acclimation.

**Table S6** Comparison of the slope and intercept of the relationships between *A*_sat_ and *V_c_*_max_ and *A*_sat_ and *J*_max_.

**Table S7** Repeated measures correlation results for relationships between *A*_sat_ and *V_c_*_max_ and *A*_sat_ and *J*_max_ for each temperature regime combination. The repeated measure was day of acclimation.


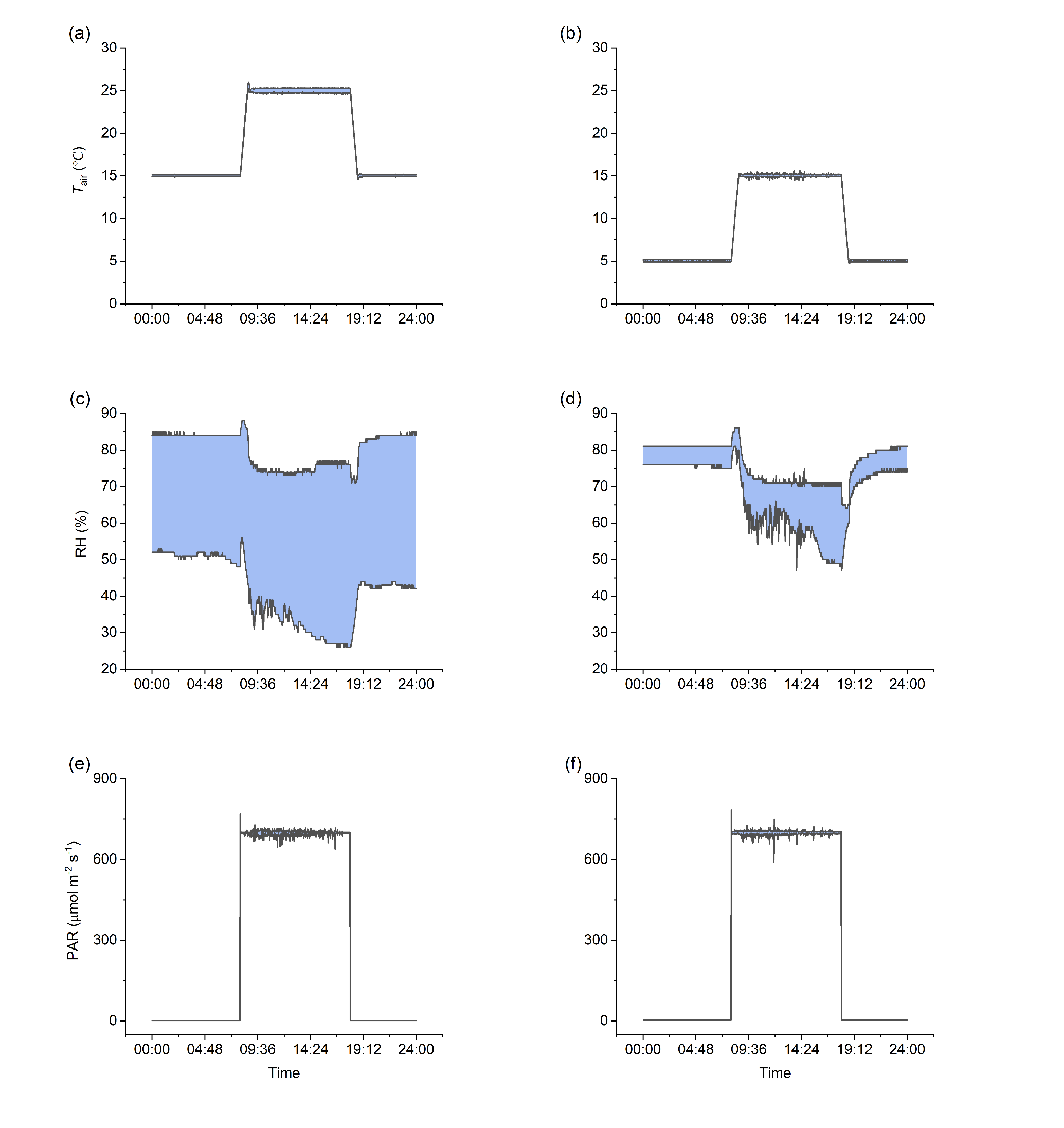


**Fig. S1** The range in climatic variables during the experiment for the 15:25℃ growth room, (a), (c) and (d), and the 5:15℃ growth room, (b), (d) and (f). *T*_air_: air temperature, RH: relative humidity, PAR: photosynthetically active radiation.


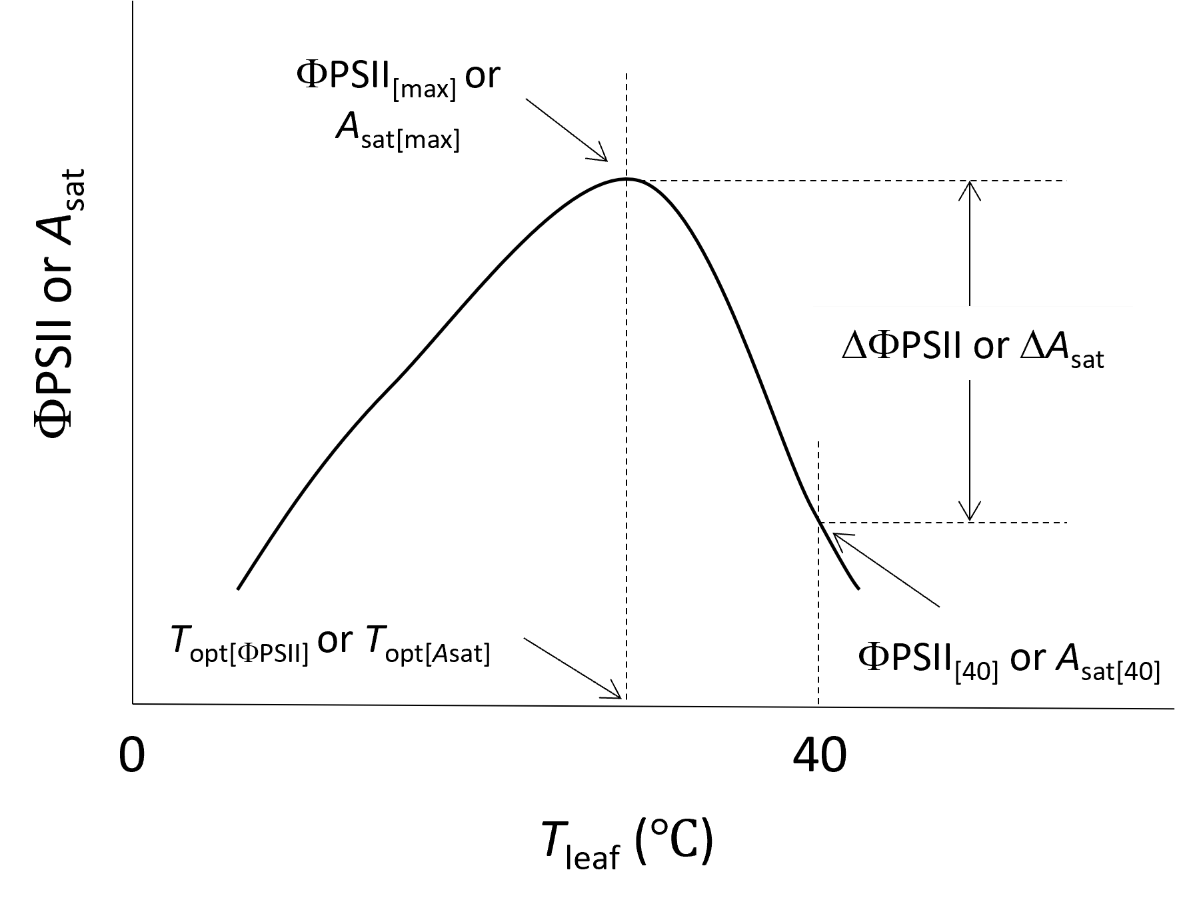


**Fig. S2** Representation of the parameters derived from the response functions of ΦPSII and *A*_sat_ to *T*_leaf_. Parameters comprise: the maximum ΦPSII (ΦPSII_[max]_) and maximum *A*_sat_ (*A*_sat[max]_) over the range of measured *T*_leaf_, the *T*_leaf_ at ΦPSII_[max]_ and *A*_sat[max]_ (*T*_opt[ΦPSII]_ and *T*_opt[_*_A_*_sat]_, respectively), the ΦPSII and *A*_sat_ at a fixed *T*_leaf_ of 40℃ (ΦPSII_[40]_ and *A*_sat[40]_ for ΦPSII and *A*_sat_, respectively) and the relative difference between ΦPSII_[max]_ and ΦPSII_[40]_ (ΔΦPSII) and the relative difference between *A*_sat[max]_ and *A*_sat[40]_ (Δ*A*_sat_).

**Table S1** Fitted parameters for the near-instantaneous temperature response functions of ΦPSII and *A*_sat_.

| **Variable** | **Accession** | **Growth temperature (℃)** | **Replicate** | **a** | **m1** | **m2** | **s1** | **s2** | **R^2^** |
| --- | --- | --- | --- | --- | --- | --- | --- | --- | --- |
| ΦPSII | CAF-Warm | 15:25 | 1 | 0.43 | 17.08 | 39.84 | 13.20 | 3.79 | 1.00 |
|  |  |  | 2 | 0.43 | 16.01 | 42.11 | 14.41 | 4.97 | 1.00 |
|  |  |  | 3 | 0.39 | 16.11 | 42.82 | 14.04 | 7.92 | 0.99 |
|  |  |  | 4 | 0.83 | 28.18 | 43.59 | 29.01 | 13.14 | 0.99 |
|  |  |  | 5 | 0.47 | 15.33 | 40.01 | 18.64 | 4.45 | 1.00 |
|  |  |  | Average | 0.42 | 15.57 | 45.52 | 14.98 | 5.46 | 0.93 |
|  |  | 5:15 | 1 | 0.28 | 10.98 | 40.07 | 10.48 | 2.88 | 0.84 |
|  |  |  | 2 | 0.44 | 14.46 | 37.92 | 18.47 | 6.29 | 0.99 |
|  |  |  | 3 | 0.71 | 21.32 | 37.51 | 21.95 | 14.98 | 0.84 |
|  |  |  | 4 | 0.67 | 20.27 | 36.32 | 21.30 | 14.57 | 0.65 |
|  |  |  | Average | 0.90 | 30.40 | 40.51 | 31.71 | 12.97 | 0.89 |
|  | CAF-Cool | 15:25 | 1 | 0.38 | 23.00 | 42.00 | 22.00 | 8.20 | 0.95 |
|  |  |  | 2 | 0.35 | 22.00 | 42.00 | 19.31 | 4.42 | 0.89 |
|  |  |  | 3 | 0.33 | 23.00 | 42.00 | 23.22 | 8.20 | 0.98 |
|  |  |  | 4 | 0.30 | 18.27 | 41.75 | 17.54 | 6.86 | 0.99 |
|  |  |  | 5 | 0.26 | 15.69 | 43.11 | 12.65 | 3.92 | 0.94 |
|  |  |  | Average | 0.25 | 15.39 | 43.28 | 12.93 | 3.98 | 0.83 |
|  |  | 5:15 | 1 | 0.45 | 14.38 | 39.22 | 14.45 | 9.00 | 0.96 |
|  |  |  | 2 | 0.41 | 11.93 | 40.73 | 10.43 | 5.65 | 0.93 |
|  |  |  | 3 | 0.37 | 12.11 | 41.46 | 9.71 | 4.69 | 0.95 |
|  |  |  | 4 | 0.51 | 17.13 | 37.91 | 21.20 | 7.87 | 1.00 |
|  |  |  | 5 | 0.40 | 16.47 | 39.71 | 17.14 | 6.19 | 0.99 |
|  |  |  | Average | 0.39 | 12.98 | 40.10 | 13.32 | 6.49 | 0.92 |
| *A*_sat_ | CAF-Warm | 15:25 | 1 | 964.1 | 50.00 | 48.40 | 29.66 | 22.56 | 0.99 |
|  |  |  | 2 | 291.7 | 50.00 | 47.21 | 38.61 | 17.26 | 0.93 |
|  |  |  | 3 | 248.9 | 50.00 | 48.18 | 43.02 | 20.22 | 0.98 |
|  |  |  | 4 | 115.0 | 50.00 | 44.69 | 69.31 | 10.44 | 0.96 |
|  |  |  | 5 | 84.2 | 27.37 | 41.29 | 52.49 | 8.35 | 0.97 |
|  |  |  | Average | 110.0 | 36.26 | 42.57 | 46.97 | 10.71 | 0.93 |
|  |  | 5:15 | 1 | 138.6 | 50.00 | 42.95 | 51.86 | 13.93 | 1.00 |
|  |  |  | 2 | 75.6 | 30.00 | 39.68 | 74.89 | 8.00 | 0.99 |
|  |  |  | 3 | 53.0 | 15.00 | 40.00 | 104.40 | 9.97 | 0.90 |
|  |  |  | 4 | 254.8 | 50.00 | 46.04 | 48.17 | 27.04 | 0.97 |
|  |  |  | Average | 55.65 | 50.00 | 41.56 | 956.04 | 8.38 | 0.87 |
|  | CAF-Cool | 15:25 | 1 | 122.8 | 50.00 | 45.60 | 48.48 | 12.55 | 0.89 |
|  |  |  | 2 | 53.1 | 50.00 | 44.06 | 123.36 | 3.69 | 0.83 |
|  |  |  | 3 | 62.0 | 50.00 | 44.68 | 81.43 | 7.38 | 0.88 |
|  |  |  | 4 | 7803.8 | 48.14 | 48.10 | 29.21 | 28.78 | 0.90 |
|  |  |  | 5 | 136.3 | 50.00 | 45.99 | 47.38 | 14.40 | 0.96 |
|  |  |  | Average | 110.0 | 66.04 | 49.11 | 69.81 | 14.65 | 0.73 |
|  |  | 5:15 | 1 | 778.8 | 50.00 | 48.44 | 36.06 | 27.56 | 0.94 |
|  |  |  | 2 | 143.2 | 50.00 | 44.78 | 73.83 | 16.53 | 0.85 |
|  |  |  | 3 | 999.5 | 35.00 | 35.93 | 25.00 | 22.57 | 0.84 |
|  |  |  | 4 | 97.0 | 28.32 | 36.04 | 38.30 | 12.35 | 0.91 |
|  |  |  | 5 | 102.1 | 50.00 | 43.62 | 69.43 | 10.46 | 0.97 |
|  |  |  | Average | 110.0 | 65.97 | 45.18 | 122.86 | 12.34 | 0.89 |

Note that one plant from accession CAF-Warm grown at 5:15℃ died prior to measurements.





**Fig. S3** First derivatives of the temperature response functions of ΦPSII and *A*_sat_ in relation to *T*_leaf_. Thick lines are the average responses of all plants per temperature regime, thin lines are the individual responses of each plant.


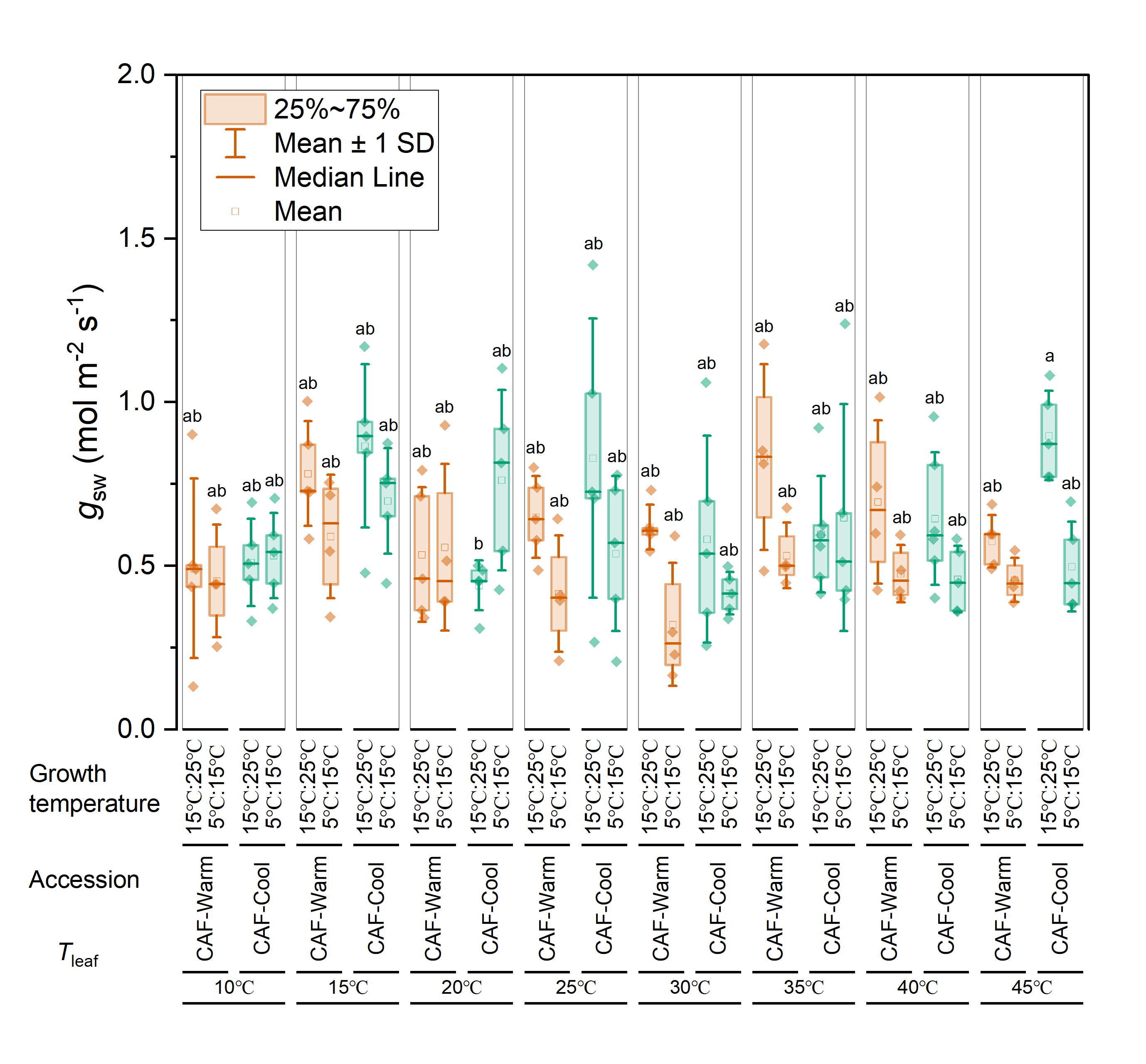


**Fig. S4** Response of *g*_sw_ to *T*_leaf_ for each accession and growth temperature. Different letters indicate significant differences between combinations of different accessions, growth temperatures and leaf temperatures (*T*_leaf_) (linear mixed models, *P* < 0.05).

Table **S2** Linear mixed effect model results for the near-instantaneous response of *g*_sw_ to *T*_leaf_. df: degrees of freedom.

| Factor | df | *F*-value | *P*-value |
| --- | --- | --- | --- |
| Accession | 1 | 0.789 | 0.3869 |
| Growth temperature | 1 | 2.139 | 0.1466 |
| *T*_leaf_ | 7 | 3.975 | **0.007**** |
| Accession x growth temperature | 1 | 2.136 | 0.1470 |
| Accession x *T*_leaf_ | 7 | 1.167 | 0.3280 |
| Growth temperature x *T*_leaf_ | 7 | 3.681 | **0.0014**** |
| Accession x growth temperature x *T*_leaf_ | 7 | 1.515 | 0.1704 |





**Fig. S5** *T*_opt[ΦPSII]_ **(a)**, *T*_opt[_*_A_*_sat]_, **(b)**, ΦPSII_[max]_ **(c)** and *A*_sat[max]_ **(d)** in relation to the growth temperature for each accession. Different letters indicate significant differences between accessions and growth temperatures (Post hoc Tukey’s HSD tests, *P* < 0.05). Refer to Fig. **S1** for a definition of the variables.





**Fig. S6** ΦPSII and *A*_sat_ recorded at a *T*_leaf_ of 40℃ (ΦPSII_[40]_ **(a)** and *A*_sat[40]_ **(b)**, respectively) and the change in ΦPSII and *A*_sat_ between ΦPSII_[max]_ and *A*_sat[max]_ (ΔΦPSII **(c)** and Δ*A*_sat_ **(d)**, respectively) in relation to the growth temperature (15:25℃ and 5:15℃) for each accession. Different letters indicate significant differences between accessions and growth temperatures (Post hoc Tukey’s HSD tests, *P* < 0.05). Refer to Fig. **S1** for a definition of the variables.

**Table S3** Photosynthetic variables derived from relationships between *A*_sat_ and *C*_i_. Switch refers to the number of days following the switch to the acclimation temperature.

| **Accession** | **Replicate** | **Growth temperature (℃)** | **Acclimation temperature (℃)** | **Switch** | ***V_c_*_max_ (µmol m^-2^ s^-1^)** | ***J*_max_ (µmol m^-2^ s^-1^)** | ***V_c_*_max_25 (µmol m^-2^ s^-1^)** | ***J*_max_25 (µmol m^-2^ s^-1^)** |
| --- | --- | --- | --- | --- | --- | --- | --- | --- |
| CAF006 | 1 | 15:25 | 15:25 | 1 | 120.7 | 237.1 | 120.7 | 237.1 |
| CAF006 | 2 | 15:25 | 15:25 | 1 | 130.9 | 261.5 | 130.9 | 261.5 |
| CAF006 | 3 | 15:25 | 15:25 | 1 | 135.8 | 259.9 | 135.8 | 259.9 |
| CAF006 | 4 | 15:25 | 15:25 | 1 | 133.8 | 246.9 | 133.8 | 246.9 |
| CAF006 | 5 | 15:25 | 15:25 | 1 | 140.1 | 259.7 | 140.1 | 259.7 |
| CAF006 | 1 | 15:25 | 15:25 | 8 | 137.9 | 316.5 | 137.9 | 316.5 |
| CAF006 | 2 | 15:25 | 15:25 | 8 | 161.1 | 303.3 | 161.1 | 303.3 |
| CAF006 | 3 | 15:25 | 15:25 | 8 | 117.1 | 214.6 | 117.1 | 214.6 |
| CAF006 | 4 | 15:25 | 15:25 | 8 | 137.3 | 263.9 | 137.3 | 263.9 |
| CAF006 | 5 | 15:25 | 15:25 | 8 | 124.4 | 281.7 | 124.4 | 281.7 |
| CAF0216 | 1 | 15:25 | 15:25 | 1 | 122.1 | 228.0 | 122.1 | 228.0 |
| CAF0216 | 2 | 15:25 | 15:25 | 1 | 119.8 | 214.4 | 119.8 | 214.4 |
| CAF0216 | 3 | 15:25 | 15:25 | 1 | 125.5 | 240.8 | 125.5 | 240.8 |
| CAF0216 | 4 | 15:25 | 15:25 | 1 | 142.1 | 258.0 | 142.1 | 258.0 |
| CAF0216 | 5 | 15:25 | 15:25 | 1 | 113.4 | 199.1 | 113.4 | 199.1 |
| CAF0216 | 1 | 15:25 | 15:25 | 8 | 115.3 | 241.9 | 115.3 | 241.9 |
| CAF0216 | 2 | 15:25 | 15:25 | 8 | 94.7 | 184.2 | 94.7 | 184.2 |
| CAF0216 | 3 | 15:25 | 15:25 | 8 | 85.7 | 158.8 | 85.7 | 158.8 |
| CAF0216 | 4 | 15:25 | 15:25 | 8 | 86.5 | 173.9 | 86.9 | 169.6 |
| CAF0216 | 5 | 15:25 | 15:25 | 8 | 59.1 | 131.3 | 59.1 | 131.3 |
| CAF006 | 1 | 5:15 | 15:25 | 1 | 106.7 | 282.1 | 106.7 | 282.1 |
| CAF006 | 2 | 5:15 | 15:25 | 1 | 84.8 | 185.3 | 84.8 | 185.3 |
| CAF006 | 3 | 5:15 | 15:25 | 1 | 97.6 | 242.0 | 97.6 | 242.0 |
| CAF006 | 4 | 5:15 | 15:25 | 1 | 113.9 | 267.8 | 113.9 | 267.8 |
| CAF006 | 5 | 5:15 | 15:25 | 1 | 120.9 | 245.7 | 120.9 | 245.7 |
| CAF006 | 1 | 5:15 | 15:25 | 8 | 145.9 | 286.8 | 145.9 | 286.8 |
| CAF006 | 2 | 5:15 | 15:25 | 8 | 148.5 | 290.4 | 148.5 | 290.4 |
| CAF006 | 3 | 5:15 | 15:25 | 8 | 131.2 | 254.2 | 133.0 | 253.5 |
| CAF006 | 4 | 5:15 | 15:25 | 8 | 141.3 | 242.3 | 141.3 | 242.3 |
| CAF006 | 5 | 5:15 | 15:25 | 8 | 125.7 | 260.1 | 125.7 | 260.1 |
| CAF0216 | 1 | 5:15 | 15:25 | 1 | 128.7 | 324.6 | 128.7 | 324.6 |
| CAF0216 | 2 | 5:15 | 15:25 | 1 | 153.9 | 375.7 | 153.9 | 375.7 |
| CAF0216 | 3 | 5:15 | 15:25 | 1 | 118.2 | 239.9 | 118.2 | 239.9 |
| CAF0216 | 4 | 5:15 | 15:25 | 1 | 126.3 | 330.4 | 126.3 | 330.4 |
| CAF0216 | 5 | 5:15 | 15:25 | 1 | 108.9 | 235.3 | 108.2 | 233.7 |
| CAF0216 | 1 | 5:15 | 15:25 | 8 | 130.0 | 236.4 | 130.0 | 236.4 |
| CAF0216 | 2 | 5:15 | 15:25 | 8 | 121.9 | 237.7 | 121.9 | 237.7 |
| CAF0216 | 3 | 5:15 | 15:25 | 8 | 126.7 | 244.4 | 126.7 | 244.4 |
| CAF0216 | 4 | 5:15 | 15:25 | 8 | 118.6 | 220.1 | 118.6 | 220.1 |
| CAF0216 | 5 | 5:15 | 15:25 | 8 | 80.5 | 181.5 | 80.5 | 181.5 |
| CAF006 | 1 | 15:25 | 5:15 | 1 | 49.4 | 133.7 | 157.0 | 226.9 |
| CAF006 | 2 | 15:25 | 5:15 | 1 | 51.8 | 137.6 | 164.7 | 233.4 |
| CAF006 | 3 | 15:25 | 5:15 | 1 | 61.6 | 153.9 | 195.8 | 261.2 |
| CAF006 | 4 | 15:25 | 5:15 | 1 | 55.9 | 128.1 | 177.9 | 217.4 |
| CAF006 | 5 | 15:25 | 5:15 | 1 | 62.4 | 146.4 | 198.5 | 248.5 |
| CAF006 | 1 | 15:25 | 5:15 | 8 | 61.1 | 184.2 | 194.4 | 312.6 |
| CAF006 | 2 | 15:25 | 5:15 | 8 | 60.2 | 194.0 | 169.5 | 309.0 |
| CAF006 | 3 | 15:25 | 5:15 | 8 | 51.3 | 154.4 | 163.1 | 262.0 |
| CAF006 | 4 | 15:25 | 5:15 | 8 | 63.3 | 167.6 | 201.4 | 284.4 |
| CAF006 | 5 | 15:25 | 5:15 | 8 | 60.0 | 167.6 | 189.8 | 281.9 |
| CAF0216 | 1 | 15:25 | 5:15 | 1 | 51.5 | 135.1 | 163.7 | 229.3 |
| CAF0216 | 2 | 15:25 | 5:15 | 1 | 55.7 | 150.1 | 177.1 | 254.7 |
| CAF0216 | 3 | 15:25 | 5:15 | 1 | 61.8 | 157.3 | 196.6 | 266.9 |
| CAF0216 | 4 | 15:25 | 5:15 | 1 | 58.4 | 127.6 | 185.8 | 216.5 |
| CAF0216 | 5 | 15:25 | 5:15 | 1 | 48.0 | 111.4 | 152.7 | 189.0 |
| CAF0216 | 1 | 15:25 | 5:15 | 8 | 48.7 | 159.7 | 154.8 | 271.0 |
| CAF0216 | 2 | 15:25 | 5:15 | 8 | 62.6 | 177.7 | 199.2 | 301.5 |
| CAF0216 | 3 | 15:25 | 5:15 | 8 | 62.1 | 167.1 | 197.5 | 283.5 |
| CAF0216 | 4 | 15:25 | 5:15 | 8 | 43.1 | 123.5 | 141.1 | 210.5 |
| CAF0216 | 5 | 15:25 | 5:15 | 8 | 35.5 | 104.7 | 112.9 | 177.7 |
| CAF006 | 1 | 5:15 | 5:15 | 1 | 58.9 | 195.1 | 187.2 | 331.0 |
| CAF006 | 2 | 5:15 | 5:15 | 1 | 48.4 | 142.9 | 159.9 | 249.8 |
| CAF006 | 3 | 5:15 | 5:15 | 1 | 67.0 | 186.0 | 212.9 | 315.7 |
| CAF006 | 4 | 5:15 | 5:15 | 1 | 49.0 | 142.7 | 155.7 | 242.2 |
| CAF006 | 5 | 5:15 | 5:15 | 1 | 51.0 | 160.2 | 162.0 | 271.7 |
| CAF006 | 1 | 5:15 | 5:15 | 8 | 58.2 | 169.5 | 185.1 | 287.6 |
| CAF006 | 2 | 5:15 | 5:15 | 8 | 63.8 | 194.1 | 204.1 | 327.1 |
| CAF006 | 3 | 5:15 | 5:15 | 8 | 45.8 | 121.2 | 145.5 | 205.7 |
| CAF006 | 4 | 5:15 | 5:15 | 8 | 45.9 | 123.1 | 146.0 | 208.8 |
| CAF006 | 5 | 5:15 | 5:15 | 8 | 59.1 | 161.9 | 188.0 | 274.8 |
| CAF0216 | 1 | 5:15 | 5:15 | 1 | 72.5 | 220.7 | 230.5 | 374.5 |
| CAF0216 | 2 | 5:15 | 5:15 | 1 | 55.7 | 160.6 | 179.0 | 276.6 |
| CAF0216 | 3 | 5:15 | 5:15 | 1 | 62.9 | 170.6 | 200.0 | 289.4 |
| CAF0216 | 4 | 5:15 | 5:15 | 1 | 62.0 | 165.3 | 197.0 | 280.4 |
| CAF0216 | 5 | 5:15 | 5:15 | 1 | 50.3 | 138.9 | 160.0 | 235.7 |
| CAF0216 | 1 | 5:15 | 5:15 | 8 | 70.1 | 199.6 | 223.0 | 338.7 |
| CAF0216 | 2 | 5:15 | 5:15 | 8 | 71.3 | 210.4 | 225.3 | 355.4 |
| CAF0216 | 3 | 5:15 | 5:15 | 8 | 59.8 | 161.7 | 190.2 | 274.4 |
| CAF0216 | 4 | 5:15 | 5:15 | 8 | 59.7 | 160.3 | 189.9 | 272.0 |
| CAF0216 | 5 | 5:15 | 5:15 | 8 | 51.5 | 142.2 | 163.6 | 241.3 |

**Table S4** Linear mixed effect model results for *V_c_*_max_, *V_c_*_max_25, *J*_max_, *J*_max_25, *J*_max_/*V_c_*_max_ and *J*_max_25/*V_c_*_max_25. df: degrees of freedom.

| Factor | df | *F*-value | *P*-value |
| --- | --- | --- | --- |
| *V_c_*_max_ |  |  |  |
| Accession | 1 | 5.296 | **0.0280*** |
| Acclimation temperature | 3 | 177.731 | **<0.001***** |
| Acclimation day | 1 | 0.671 | 0.4190 |
| Accession x acclimation temperature | 3 | 8.158 | **0.004**** |
| Acclimation day x accession | 1 | 23.696 | **<0.001***** |
| Acclimation day x acclimation temperature | 3 | 5.822 | **0.0028**** |
| Acclimation day x acclimation temperature x accession | 3 | 6.864 | **0.0011**** |
| *V_c_*_max_25 |  |  |  |
| Accession | 1 | 0.957 | 0.3353 |
| Acclimation temperature | 3 | 46.931 | **<0.001***** |
| Acclimation day | 1 | 0.101 | 0.7527 |
| Accession x acclimation temperature | 3 | 4.605 | **0.0087**** |
| Acclimation day x accession | 1 | 9.218 | **0.0048**** |
| Acclimation day x acclimation temperature | 3 | 2.054 | 0.1266 |
| Acclimation day x acclimation temperature x accession | 3 | 2.360 | 0.0905 |
| *J*_max_ |  |  |  |
| Accession | 1 | 4.0983 | 0.0513 |
| Acclimation temperature | 3 | 53.4007 | **<0.001***** |
| Acclimation day | 1 | 1.2459 | 0.2729 |
| Accession x acclimation temperature | 3 | 6.4146 | **0.0016**** |
| Acclimation day x accession | 1 | 15.2818 | **0.001**** |
| Acclimation day x acclimation temperature | 3 | 3.1128 | **0.0404*** |
| Acclimation day x acclimation temperature x accession | 3 | 5.0937 | **0.0055**** |
| *J*_max_25 |  |  |  |
| Accession | 1 | 2.3196 | 0.1376 |
| Acclimation temperature | 3 | 4.5963 | **0.0087**** |
| Acclimation day | 1 | 0.2261 | 0.6377 |
| Accession x acclimation temperature | 3 | 4.3281 | **0.0114*** |
| Acclimation day x accession | 1 | 10.5004 | **0.0028**** |
| Acclimation day x acclimation temperature | 3 | 3.5251 | **0.0263*** |
| Acclimation day x acclimation temperature x accession | 3 | 3.9089 | **0.0177*** |
| *J*_max_/*V_c_*_max_ |  |  |  |
| Accession | 1 | 1.006 | 0.3234 |
| Acclimation temperature | 3 | 101.924 | **<0.001***** |
| Acclimation day | 1 | 0.514 | 0.4786 |
| Accession x acclimation temperature | 3 | 0.445 | 0.7225 |
| Acclimation day x accession | 1 | 0.802 | 0.3773 |
| Acclimation day x acclimation temperature | 3 | 21.877 | **<0.001***** |
| Acclimation day x acclimation temperature x accession | 3 | 0.294 | 0.8296 |
| *J*_max_25/*V_c_*_max_25 |  |  |  |
| Accession | 1 | 0.918 | 0.3451 |
| Acclimation temperature | 3 | 104.038 | **<0.001***** |
| Acclimation day | 1 | 0.659 | 0.4231 |
| Accession x acclimation temperature | 3 | 0.408 | 0.7485 |
| Acclimation day x accession | 1 | 0.325 | 0.5727 |
| Acclimation day x acclimation temperature | 3 | 19.167 | **<0.001***** |
| Acclimation day x acclimation temperature x accession | 3 | 0.153 | 0.9267 |

*0.01≤*P*≤0.05, **0.001≤*P*<0.01, ****P*<0.001


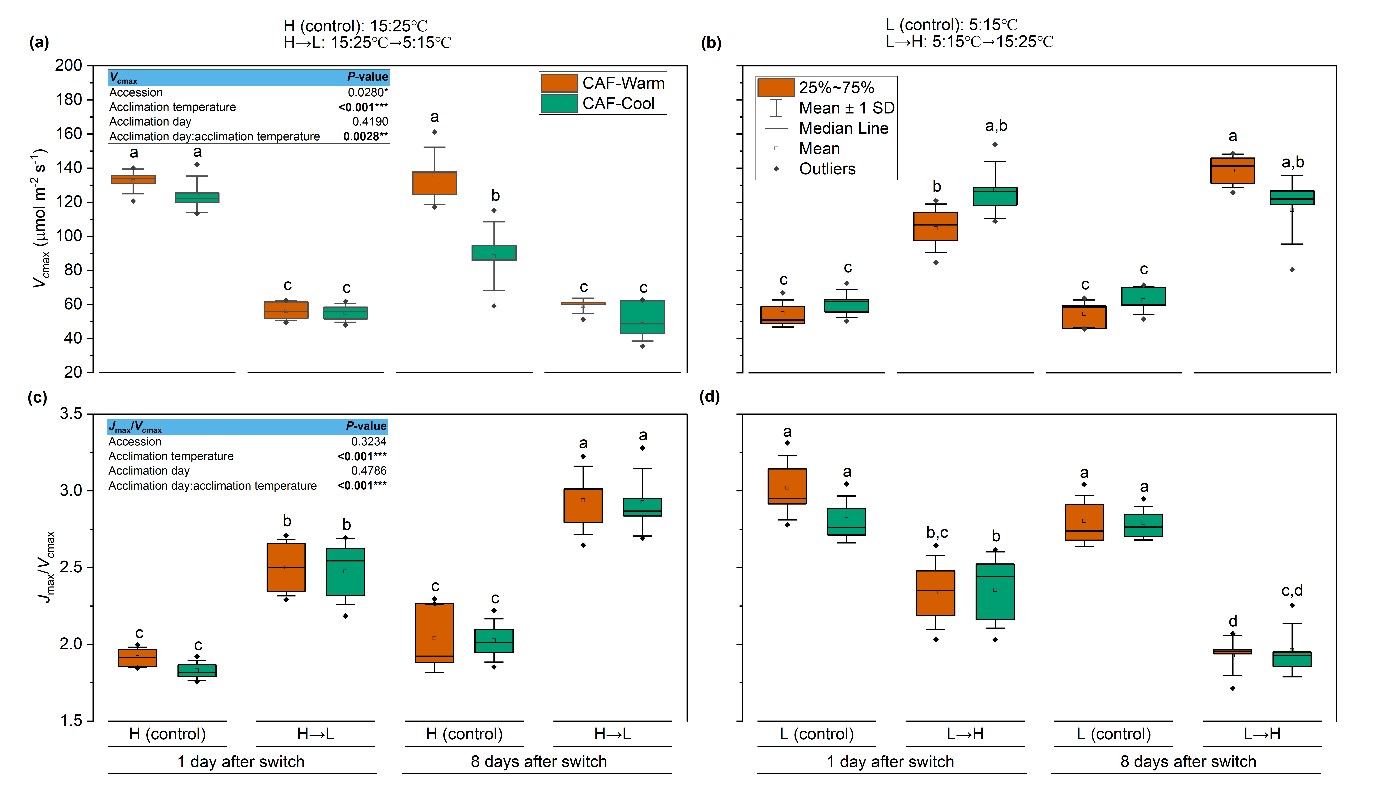


**Fig. S7** *V_c_*_max_ **(a, b)** and *J*_max_/*V_c_*_max_ **(c, d)** of each accession after 1-day and 8-days of temperature acclimation compared to control plants. The *V_c_*_max_ and *J*_max_ data reflect measurements at the daytime air temperature (15℃ or 25℃). Different letters in panels indicate significant differences between combinations of different accessions, acclimation days and acclimation temperatures (linear mixed models, *P* < 0.05). Between factor comparisons across the entire dataset from the linear mixed models are summarised in the inset tables, with further details of the model results shown in the Table **S3**.


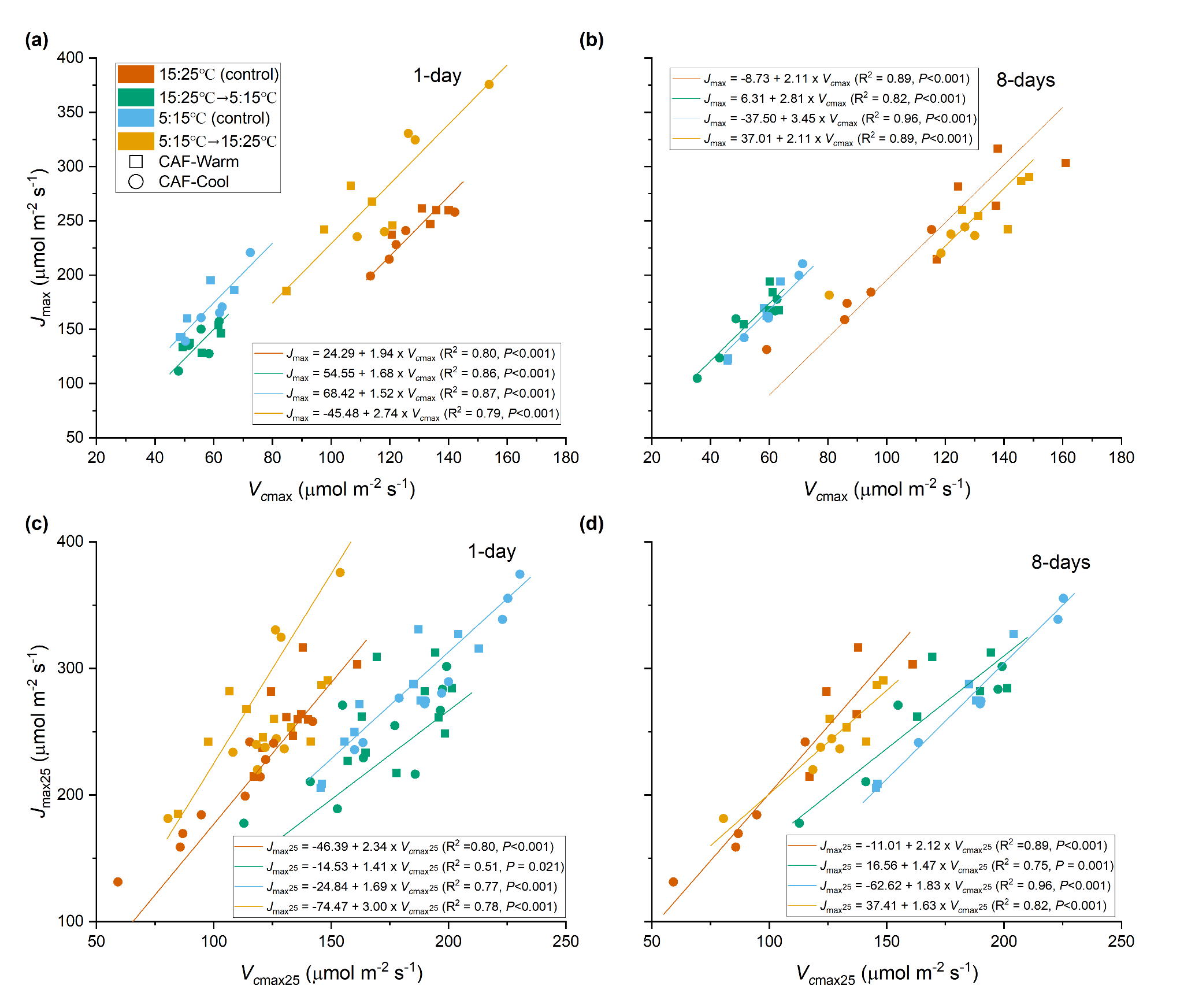


**Fig. S8** Relationships between *J*_max_ and *V_c_*_max_ after 1-day of temperature acclimation **(a)** and 8-days of temperature acclimation **(b)**, and relationships between *J*_max_25 and *V_c_*_max_25 after 1-day **(c)** and 8-days **(d)** of temperature acclimation. Fitted lines are SMA regression models. Model comparisons are shown in Table **S4**.

**Table S5** SMA test results for differences in the slope and intercept of the relationships between *J*_max_ and *V_c_*_max_ and *J*_max_25 and *V_c_*_max_25 after 1-day and 8-days of temperature acclimation.

| Variables | Day of acclimation | Group 1 | Group 2 | *P*-value (slope) | *P*-value (Intercept) | Figure |
| --- | --- | --- | --- | --- | --- | --- |
| *J*_max_*~V_c_*_max_ | 1 | 25℃ | 15→25℃ | 0.223 | **<0.001***** | Fig. S7a |
|  |  | 15℃ | 25→15℃ | 0.572 | **<0.001***** |  |
|  | 8 | 25℃ | 15→25℃ | 0.212 | 0.248 | Fig. S7b |
|  |  | 15℃ | 25→15℃ | 0.239 | 0.113 |  |
| *J*_max_25*~V_c_*_max_25 | 1 | 25℃ | 15→25℃ | 0.221 | **<0.001***** | Fig. S7c |
|  |  | 15℃ | 25→15℃ | 0.555 | **<0.001***** |  |
|  | 8 | 25℃ | 15→25℃ | 0.193 | 0.251 | Fig. S7d |
|  |  | 15℃ | 25→15℃ | 0.264 | 0.110 |  |

Note, for a simplified presentation in the table, 25℃ is the 15:25℃ regime, 15℃ is the 5:15℃ regime, 25→15℃ is the 15:25→5:15℃ regime and 25→15℃ is the 15:25→5:15℃ regime.

****P*<0.001

**Table S6** Comparison of the slope and intercept of the relationships between *A*_sat_ and *V_c_*_max25_.

| x-variable | Accession | Group 1 | Group 2 | *P*-value (slope) | *P*-value (Intercept) | Figure |
| --- | --- | --- | --- | --- | --- | --- |
| *V_c_*_max_ | CAF-Warm | 25℃ and 15→25℃ | 15℃ and 25→15℃ | **0.002**** | NA | Fig. 5a |
|  | CAF-Cool | 25℃ and 15→25℃ | 15℃ and 25→15℃ | **0.304** | **<0.001***** | Fig. 5b |

Note, for a simplified presentation in the table, 25℃ is the 15:25℃ regime, 15℃ is the 5:15℃ regime, 25→15℃ is the 15:25→5:15℃ regime and 25→15℃ is the 15:25→5:15℃ regime.

**0.001≤*P*≤0.01, ****P*<0.001

**Table S7** Repeated measures correlation results for relationships between *A*_sat_ and *V_c_*_max25_ for each temperature regime combination. The repeated measure was day of acclimation.

| Variables | Accession | Group | R | df | *P*-value | Figure |
| --- | --- | --- | --- | --- | --- | --- |
| *A*_sat_*~V_c_*_max25_ | CAF-Warm | All plants | -0.22034 | 37 | 0.17 | Fig. 5a |
|  |  | 25℃ | 0.69049 | 7 | **0.0395*** |  |
|  |  | 25→15℃ | 0.71710 | 7 | **0.0297*** |  |
|  |  | 15℃ | 0.96701 | 7 | **<0.001***** |  |
|  |  | 15→25℃ | 0.55878 | 7 | 0.1178 |  |
|  |  | 25℃ and 15→25℃ | 0.79886 | 17 | **<0.001***** |  |
|  |  | 15℃ and 25→15℃ | 0.79999 | 17 | **<0.001***** |  |
|  | CAF-Cool | All plants | 0.13833 | 36 | 0.4075 | Fig. 5b |
|  |  | 25℃ | 0.81564 | 6 | **0.0136*** |  |
|  |  | 25→15℃ | 0.86394 | 7 | **0.0027**** |  |
|  |  | 15℃ | 0.72010 | 7 | **0.0287*** |  |
|  |  | 15→25℃ | 0.83847 | 7 | **0.0047**** |  |
|  |  | 25℃ and 15→25℃ | 0.77097 | 16 | **<0.001***** |  |
|  |  | 15℃ and 25→15℃ | 0.81537 | 17 | **<0.001***** |  |

Note, for a simplified presentation in the table, 25℃ is the 15:25℃ regime, 15℃ is the 5:15℃ regime, 25→15℃ is the 15:25→5:15℃ regime and 25→15℃ is the 15:25→5:15℃ regime.

*0.01≤*P*≤0.05, **0.001≤*P*<0.01, ****P*<0.001
